# Correction: A Transposable Element within the Non-canonical Telomerase RNA of *Arabidopsis thaliana* Modulates Telomerase in Response to DNA Damage

**DOI:** 10.1371/journal.pgen.1005417

**Published:** 2015-08-18

**Authors:** The PLOS Genetics Staff

## Notice of Republication

This article was republished on June 29, 2015, to correct an error in the title that was introduced during the typesetting process. The publisher apologizes for the error. Please download this article again to view the correct version. The originally published, uncorrected article and the republished, corrected article are provided here for reference.

## Supporting Information

S1 File Originally published, uncorrected article. (PDF)

S2 File Republished, corrected article. (PDF)
