# Supplementary material for: A Transposable Element within the Non-canonical Telomerase RNA of Arabidopsis thaliana Modulates Telomerase in Response to DNA Damage
Source: PLoS Genet. 2015 Jun 15;11(6):e1005281. doi: 10.1371/journal.pgen.1005281 (PMC4468102; doi:10.1371/journal.pgen.1005281)
Supplement: S1 Table — (PDF) [file pgen.1005281.s001.pdf]

Accession	Predicted DRE status	Genotyped DRE status
**Col-0**	Full	Full
**Ler-0**	None	None
**Aa_0**	Partial	None
**Ang_0**	Partial	None
**Co_1**	Partial	None
**Ei_2**	Partial	None
**Gu_0**	Partial	None
**Je_0**	Partial	None
**Jl_3**	Partial	None
**Knox_18**	Partial	None
**Ra_0**	Partial	None
**Ber**	Full	Full
**Bik_1**	Full	Full
**Krot_0**	Full	Full
**Nc_1**	Full	Full
**Nok_3**	Full	Full
**Qar_8a**	Full	Full
**Ws_2**	Full	Full

Supplemental Table 1
