# Supplementary figures and images for: A Transposable Element within the Non-canonical Telomerase RNA of Arabidopsis thaliana Modulates Telomerase in Response to DNA Damage
Source: PLoS Genet. 2015 Jun 15;11(6):e1005281. doi: 10.1371/journal.pgen.1005281 (PMC4468102; doi:10.1371/journal.pgen.1005281)

**A**

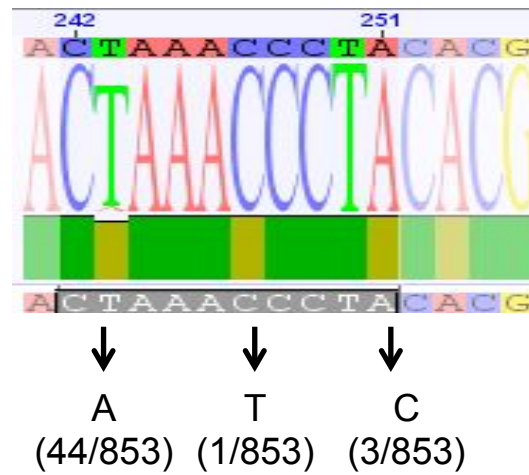

**B**

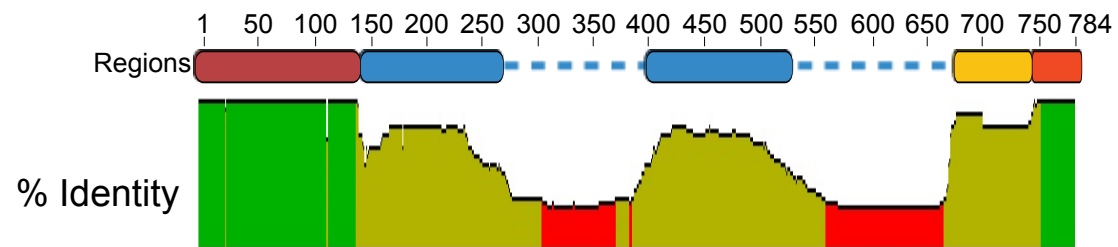

Supplemental Figure 2

Supplement: S2 Fig — (A) Screenshot of a Geneious alignment of TER1 template regions amongst 853 A. thaliana accessions (grey box). Three types of polymorphisms are observed. The arrows point to the nucleotide changes and their observed frequency. (B) Alignment of only the accessions showing partial intron loss within TER2. The green bar indicates complete nucleotide identity among accessions. Height of the yellow bars is indicative of nucleotide variation or loss of nucleotide sequence at that position. Two conserved regions in the intron (DRE) are highlighted by horizontal blue bars. The dashed line denotes two hypervariable regions. (PDF) [file pgen.1005281.s003.pdf]

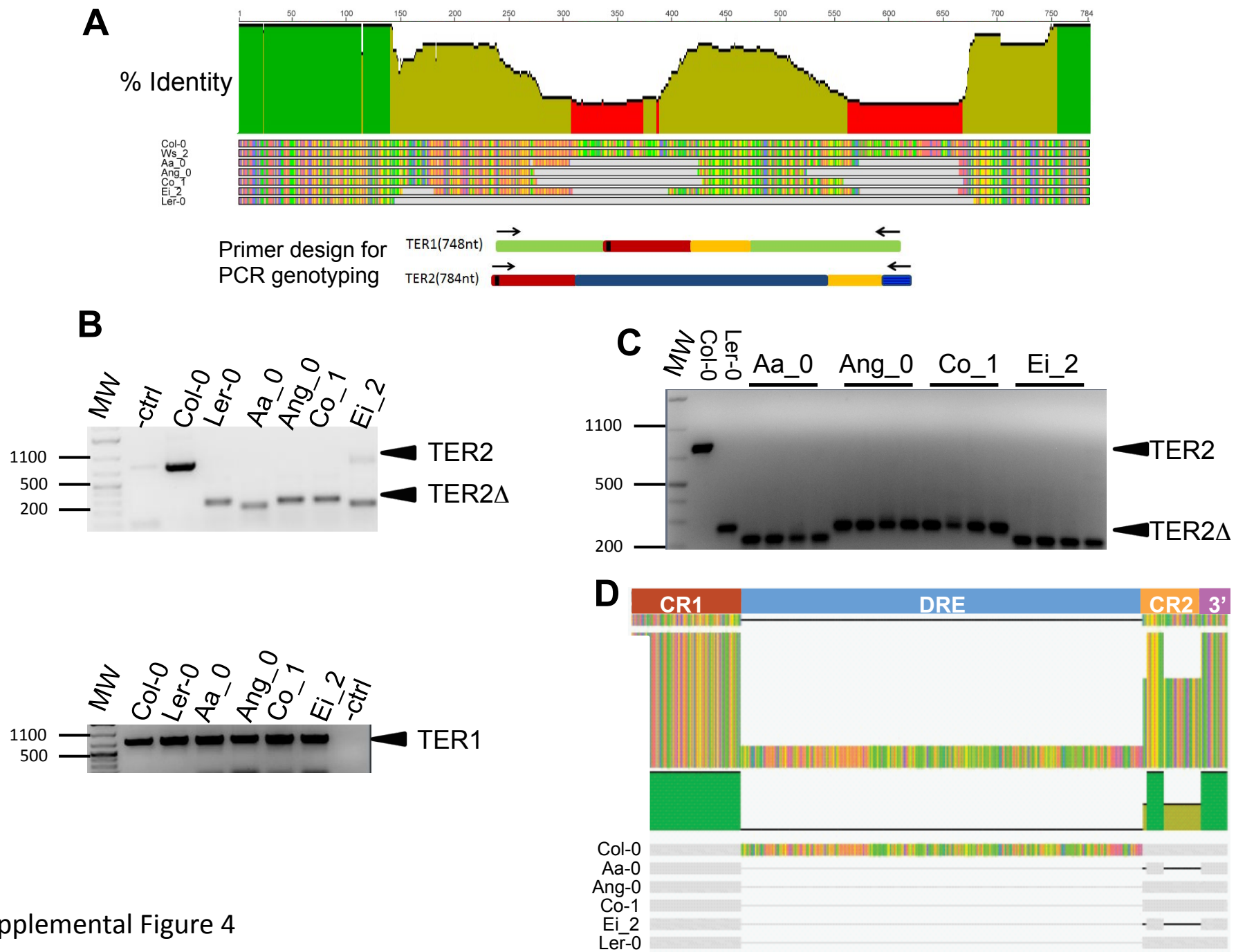

Supplemental Figure 4

Supplement: S4 Fig — (A) Schematic map of the status of the TER2 intervening sequence in different accessions. The positions of PCR primers are indicated by black arrows. (B) Genotyping results for TER1 and TER2 loci in different accessions. TER2 PCR products with the full-length DRE are expected to be ~750 bp, and PCR products lacking DRE are ~200 bp. Col-0 was used as a full-length DRE control, and Ler-0 used as complete DRE loss control. Sequence analysis of all of the TER1 PCR products confirmed accession identity. (C) TER2 DRE genotyping results in four siblings of each accession. (D) Sequencing data for TER2 genotyping PCR products in (B). The gaps in DRE and CR2 demonstrate sequence loss for these accessions. (PDF) [file pgen.1005281.s005.pdf]

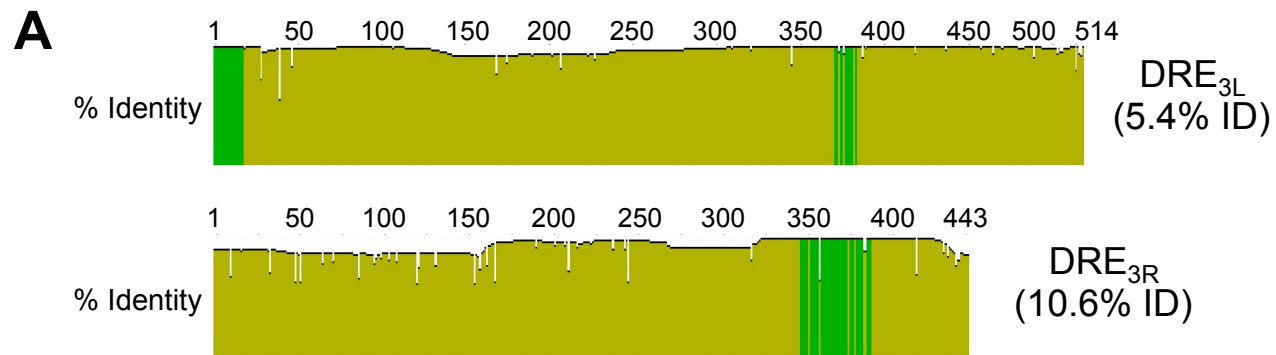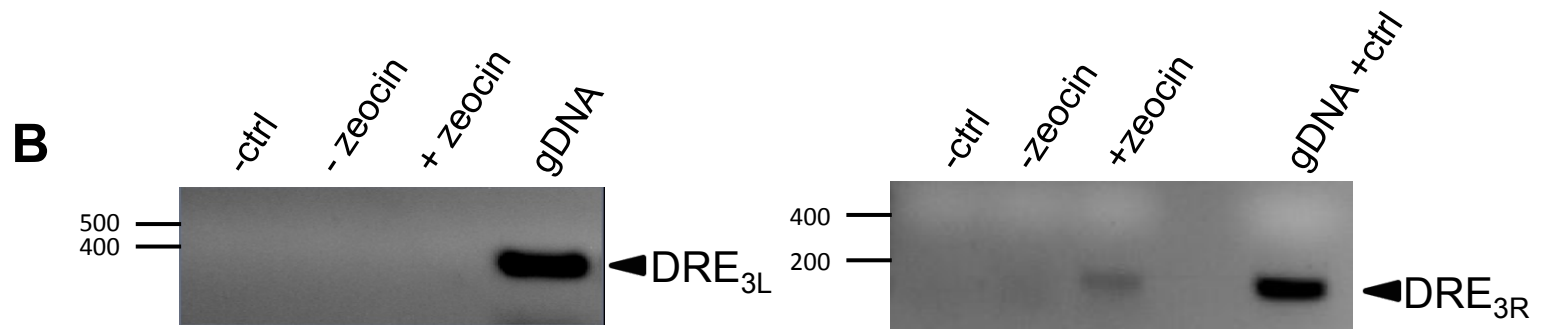

Supplemental Figure 5

Supplement: S5 Fig — (A) DRE3L and DRE3R conservation across A. thaliana ecotypes. (B) RT-PCR results for DRE3L and DRE3R with or without zeocin treatment. The expected sizes of the PCR products are highlighted by arrows. (PDF) [file pgen.1005281.s006.pdf]

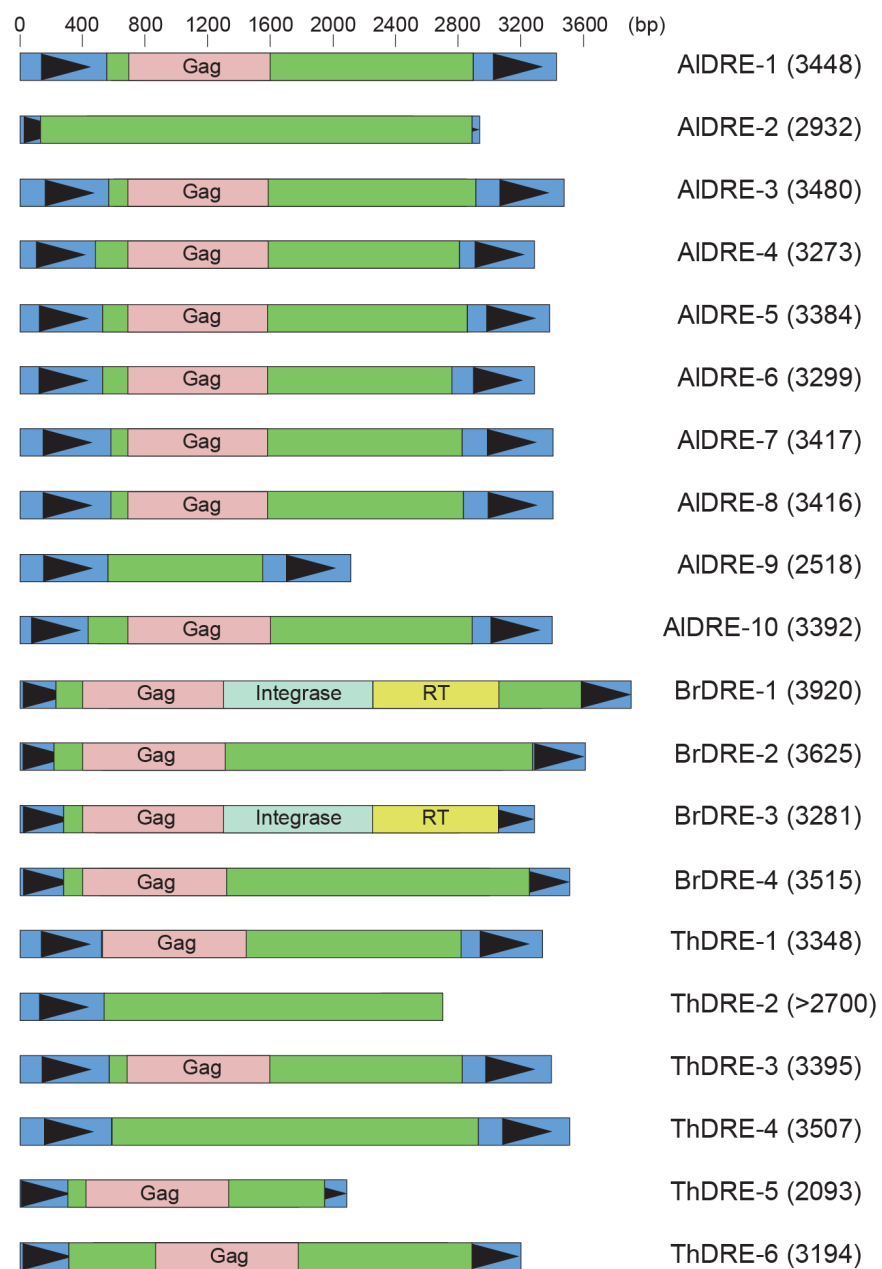

Supplemental Figure 6

Supplement: S6 Fig — Schematic representation of Brassicaceae DRE organization. Name and length (in base pairs) are on the right. LTRs (black arrows in blue background) as well as intact Gag, Integrase and RT (Reverse Transcriptase) ORFs are indicated. BrDRE-1 and BrDRE-3 encode for all intact Pol components (Integrase and RT) except for RNase. ThDRE-2 does not contain a second LTR, but shows high sequence similarity to ThDRE-1 throughout. AlDRE-2 does not contain an ORF, and shows low sequence similarity between LTRs. (PDF) [file pgen.1005281.s007.pdf]

# GEvo Genome Evolution Analysis

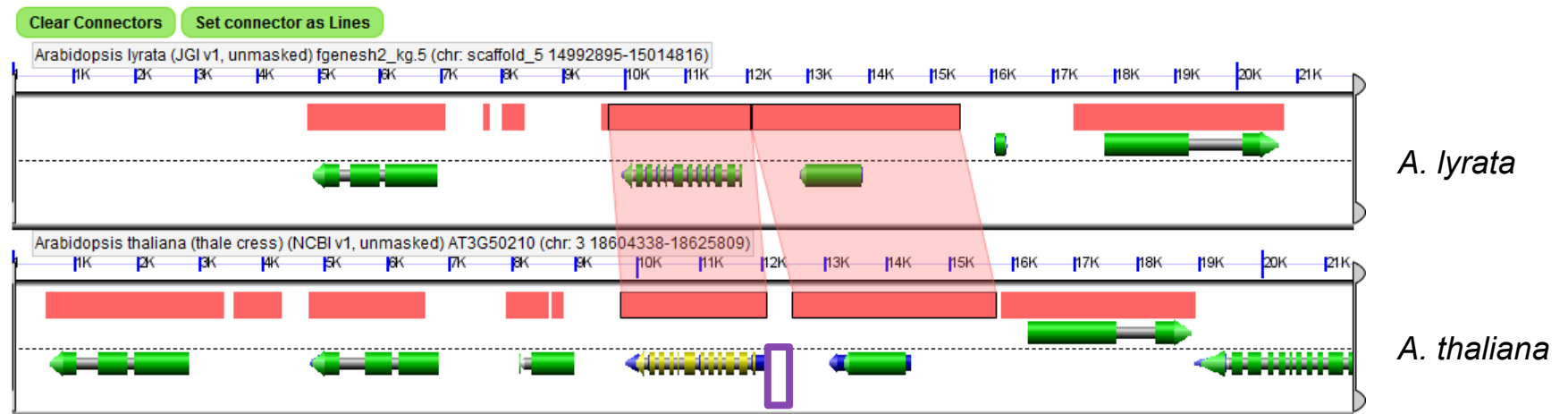

Supplemental Figure 7

Supplement: S7 Fig — CoGe (https://genomevolution.org/CoGe/) screenshot depicting synteny at the locus harboring DRE3R in A. thaliana and the closest relative, A. lyrata. Purple box indicates where the transposable element is located in A. thaliana (bottom genome). The pink rectangles denote regions of synteny between A. thaliana and A. lyrata (top genome). The gap in synteny between the two species where the transposable element resides in A. thaliana is indicative of a recent insertion. (PDF) [file pgen.1005281.s008.pdf]

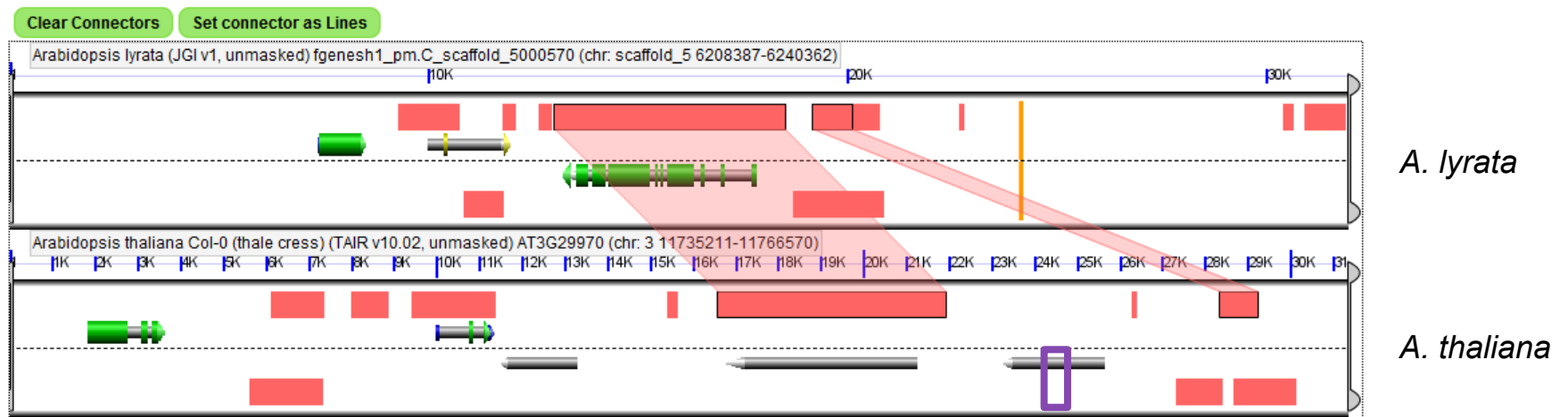

Supplemental Figure 8

Supplement: S8 Fig — CoGe screenshot of the DRE 3L locus. Purple box indicates where the transposable element is located in A. thaliana (bottom genome). The pink rectangles denote regions of synteny between A. thaliana and A. lyrata (top genome). A large section of DNA has been inserted in A. thaliana that is not present in A. lyrata. In A. thaliana, the inserted DNA includes a pseudogene (grey bars) and the DRE3L (purple box). Sequence similar to the region surrounding DRE3L is not found anywhere else in the A. lyrata or A. thaliana genomes. (PDF) [file pgen.1005281.s009.pdf]

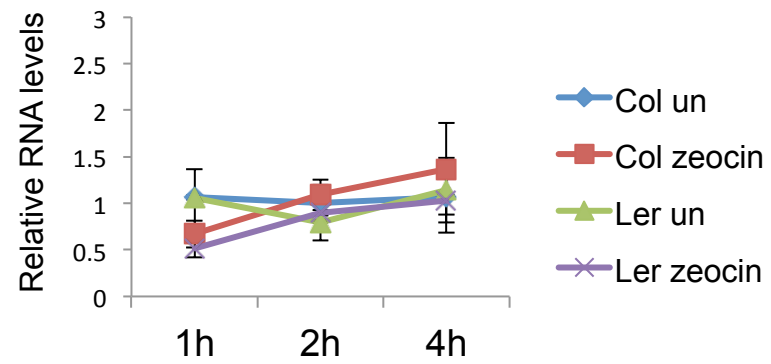

Supplemental Figure 9

Supplement: S9 Fig — qPCR results for TER1. The reaction was performed in parallel with the experiments shown in Fig 4B and 4C. (PDF) [file pgen.1005281.s010.pdf]

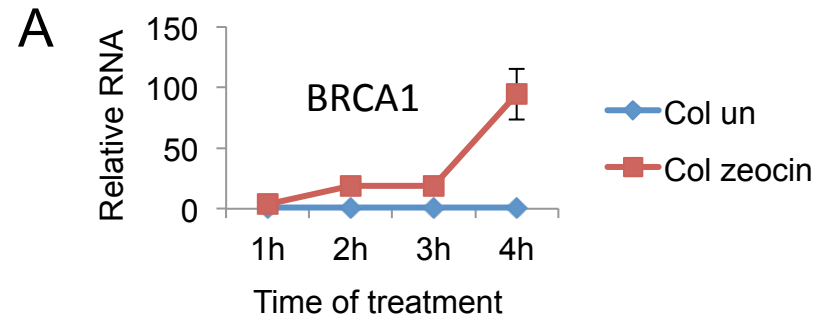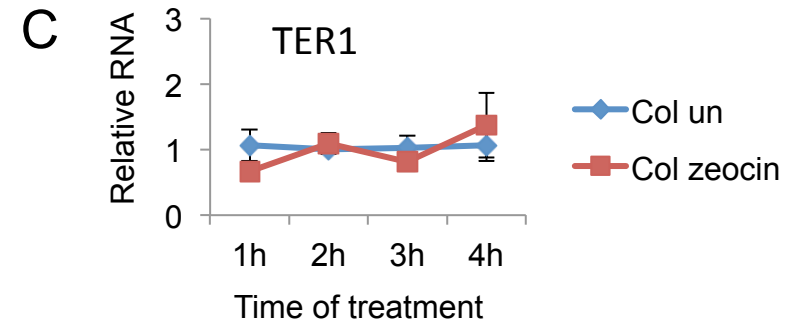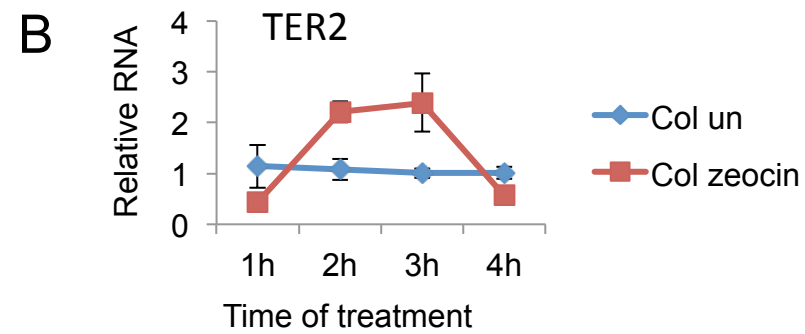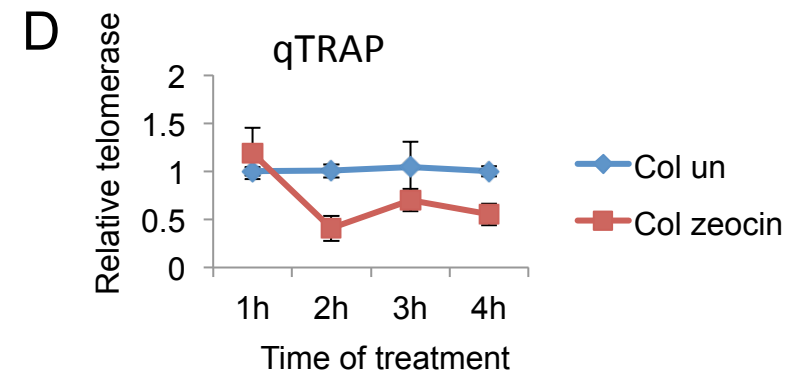

Supplemental Figure 10

Supplement: S10 Fig — The experimental design was the same as in Fig 4, with the addition of a 3 hour time point. BRCA1 (A), TER2 (B) and TER1 (C) RNA transcripts were determined by qRT-PCR. Telomerase activity was determined by qTRAP (D). The X-axis indicates time of zeocin treatment (PDF) [file pgen.1005281.s011.pdf]

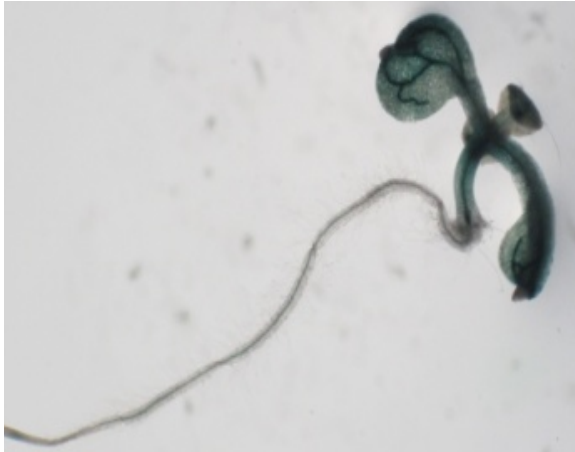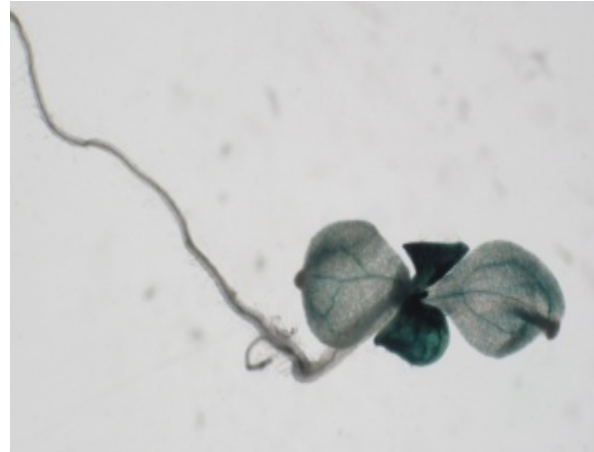

- zeocin

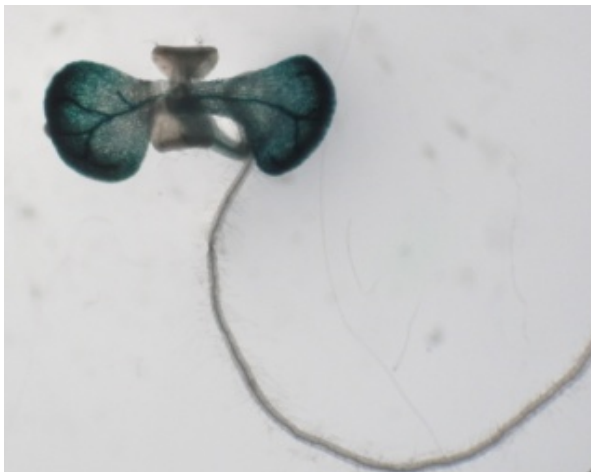

TER2::GUS in Col-0

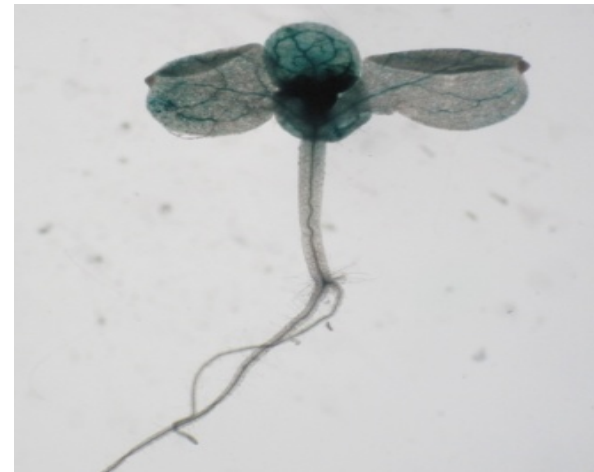

+ zeocin

TER2::GUS in Ler-0

Supplement: S11 Fig — Sequences 3kb upstream of TER2 were cloned into a vector containing the GUS gene as a reporter. The construct was transformed into both Col-0 and Ler-0. Seven day-old seedlings were treated with zeocin for 2 hours and then tested for GUS activity. (PDF) [file pgen.1005281.s012.pdf]
