# Supplementary material for: Correction: A Transposable Element within the Non-canonical Telomerase RNA of Arabidopsis thaliana Modulates Telomerase in Response to DNA Damage
Source: PLoS Genet. 2015 Aug 18;11(8):e1005417. doi: 10.1371/journal.pgen.1005417 (PMC4540318; doi:10.1371/journal.pgen.1005417)
Supplement: S1 File — (PDF) [file pgen.1005417.s001.pdf]

PLOS | GENETICS

RESEARCH ARTICLE

A Transposable Element within the Non-canonical Telomerase RNA of *Arabidopsis thaliana* Telomerase in Response to DNA Damage

Hengyi Xu, Andrew D. L. Nelson¤, Dorothy E. Shippen*

Department of Biochemistry and Biophysics, Texas A&M University, College Station, Texas, United States of America

¤ Current address: School of Plant Sciences, University of Arizona, Tucson, Arizona, United States of America
* dshippen@tamu.edu

CrossMark
click for updates

Abstract

Long noncoding RNAs (lncRNAs) have emerged as critical factors in many biological processes, but little is known about how their regulatory functions evolved. One of the best-studied lncRNAs is TER, the essential RNA template for telomerase reverse transcriptase. We previously showed that *Arabidopsis thaliana* harbors three TER isoforms: TER1, TER2 and TER2S. TER1 serves as a canonical telomere template, while TER2 is a novel negative regulator of telomerase activity, induced in response to double-strand breaks (DSBs). TER2 contains a 529 nt intervening sequence that is removed along with 36 nt at the RNA 3' terminus to generate TER2S, an RNA of unknown function. Here we investigate how *A. thaliana* TER2 acquired its regulatory function. Using data from the 1,001 Arabidopsis genomes project, we report that the intervening sequence within TER2 is derived from a transposable element termed DSB responsive element (DRE). DRE is found in the TER2 loci of most but not all *A. thaliana* accessions. By analyzing accessions with (TER2) and without DRE (TER2Δ) we demonstrate that this element is responsible for many of the unique properties of TER2, including its enhanced binding to TERT and telomerase inhibitory function. We show that DRE destabilizes TER2, and further that TER2 induction by DNA damage reflects increased RNA stability and not increased transcription. DRE-mediated changes in TER2 stability thus provide a rapid and sensitive switch to fine-tune telomerase enzyme activity. Altogether, our data shows that invasion of the TER2 locus by a small transposon converted this lncRNA into a DNA damage sensor that modulates telomerase enzyme activity in response to genome assault.

OPEN ACCESS

Citation: Xu H, Nelson ADL, Shippen DE (2015) A Transposable Element within the Non-canonical Telomerase RNA of *Arabidopsis thaliana* Telomerase in Response to DNA Damage. PLoS Genet 11(6): e1005281. doi:10.1371/journal.pgen.1005281

Editor: Jin-Qiu Zhou, Chinese Academy of Sciences, CHINA

Received: October 31, 2014

Accepted: May 14, 2015

Published: June 15, 2015

Copyright: © 2015 Xu et al. This is an open access article distributed under the terms of the Creative Commons Attribution License, which permits unrestricted use, distribution, and reproduction in any medium, provided the original author and source are credited.

Data Availability Statement: All relevant data are within the paper and its Supporting Information files.

Funding: This work was supported by the National Science Foundation MCB-1052018 to DES. The funders had no role in study design, data collection and analysis, decision to publish, or preparation of the manuscript.

Competing Interests: The authors have declared that no competing interests exist.

Author Summary

Telomerase is a highly regulated enzyme whose activity is essential for long-term cellular proliferation. In the presence of DNA double-strand breaks (DSBs), telomerase activity must be curtailed to promote faithful DNA repair. We previously showed that the flowering plant *Arabidopsis thaliana* rapidly down-regulates telomerase in response to DSBs, and further that this mode of regulation is dependent on TER2, a non-canonical telomerase RNA subunit. Here we demonstrate that the unique regulatory properties of TER2 are conveyed by a transposable element (TE) embedded in the TER2 gene. A comparison of *A*. *thaliana* accessions with and without the TE revealed that the element increases the binding affinity of TER2 for the telomerase catalytic subunit TERT relative to the canonical telomerase RNA subunit. The TE also increases TER2 turnover. In response to DSBs, TER2 is induced and accumulates in TERT containing complexes *in vivo*. Thus, invasion of a TE endows TER2 with a DNA damage sensor to rapidly and reversibly modulate enzyme activity in response to genotoxic stress. These findings provide an example of how exaptation of a TE altered the function of a long noncoding RNA. In this case, a duplicated gene (TER2) was used as the platform, and the TE as the tool to engineer a novel mode of telomerase regulation.

Introduction

The discovery of long noncoding RNA (lncRNA) has challenged the prevailing paradigm of protein-mediated regulation of gene expression and cell behavior. lncRNAs play essential roles in epigenetic regulation, stem cell biology and signal transduction and are emerging as key targets in human disease [1–3]. Unlike small regulatory RNAs (e.g. miRNAs, siRNAs), lncRNAs are not subjected to purifying selection, and as a consequence they are very poorly conserved, tending to emerge quickly and evolve swiftly [4]. Although transcriptome analyses have uncovered a vast array of lncRNAs, just a tiny fraction of these have an assigned biological function, and fewer still an ascribed molecular mechanism. Little is known about the evolutionary pathways via which lncRNAs gain new functions.

The telomerase RNA subunit TER is a lncRNA and an integral component of the telomerase enzyme. TER functions as template to direct the synthesis of telomeric DNA by the telomerase reverse transcriptase TERT. Telomerase continually synthesizes telomeric DNA in stem and germline cells to avert cellular senescence. Conversely, in cells with limited proliferation programs telomerase activity is repressed, an outcome in vertebrates that may have evolved to avert tumorigenesis [5,6]. Mechanisms of telomerase regulation are varied and complex, and include modulation of telomerase localization, recruitment to the telomere and enzymology at the chromosome terminus [7]. Within the telomerase ribonucleoprotein itself, the major target of enzyme regulation is TERT. However, TER is also implicated in telomerase control. In addition, different isoforms of core telomerase components influence telomerase behavior [8,9].

In conjunction with modulating telomerase action at natural chromosome ends, the enzyme must also be restrained from acting at sites of DNA double-strand breaks (DSBs). Barbara McClintock coined the term "chromosome healing" to describe the acquisition of telomeres on broken chromosomes in maize [10]. Although de novo telomere formation (DNTF) protects the terminus from subsequent repair activities, it leads to loss of the centromere distal chromosome fragment. Thus, DSBs must be sheltered from telomerase action to prevent gross chromosomal rearrangements and loss of heterozygosity. Multiple pathways evolved to prevent the establishment of telomeres at DSBs in yeast [11]. For example, phosphorylation of the Cdc13

telomere binding protein decreases its affinity for DSBs [12]. In addition, the Pif1 helicase is activated by DSBs, resulting in removal of telomerase from DNA [13]. Less is known about how DNTF is repressed in multicellular eukaryotes. In mammals, DSBs trigger TERT phosphorylation leading to decreased telomerase activity [14]. In addition, ionizing radiation causes transient sequestration of TERT in the nucleolus [15]. In *Arabidopsis thaliana*, a non-canonical TER represses telomerase activity in response to DSBs [16].

TER ranges in size from 150 nt in Tetrahymena to >2 kb in certain fungi, and while the nucleotide sequence is highly variable across species, core secondary and tertiary structures are conserved and essential for TER interaction with TERT and for telomerase catalysis [17–21]. TER is transcriptionally regulated in mammals [22], but the transcript is highly stable with a half-life of several days [23]. Recent data show that that 3' terminus of *Schizosaccharomyces pombe* TER is generated by an additional RNA processing step termed slicing, which involves only the first step in mRNA splicing [24,25]. Conventional introns have not been associated with TER.

Arabidopsis thaliana is unusual in that it harbors two *TER* genes, TER1 (784 nt) and TER2 (748 nt) [26]. Within TER1 and TER2, there are two regions of high similarity spanning ~219 nt termed conserved region 1 (CR1) and conserved region 2 (CR2). In TER2, CR1 and CR2 are separated by a 529 nt intervening sequence. An additional unique 36 nts lie at the 3' end of the TER2 CR2 termed 3'R. The intervening sequence and 3'R are removed *in vivo* to create a truncated isoform called $TER2_S$ [16]. Sequences flanking the intervening sequence do not adhere to consensus splice donor and acceptor sites, suggesting that removal of this element may not proceed via conventional mRNA splicing.

Although the function of $TER2_S$ is unclear, TER1 and TER2 play opposing roles in the control of telomerase enzyme activity. TER1 serves as the canonical telomere repeat template necessary for telomere length maintenance *in vivo* [26]. Plants deficient in TER1 exhibit progressive telomere shortening, and mutations in the TER1 template alter the telomere repeat sequence *in vivo*. In contrast, TER2 does not direct telomere repeat incorporation *in vivo*. Instead, this RNA negatively regulates TER1-mediated enzyme activity. Telomerase activity is elevated in plants lacking TER2, while in plants over-expressing TER2, telomerase activity is decreased and telomeres shorten [16].

TER2 is regulated by DNA damage. Under standard growth conditions, the steady state levels of TER1 and $TER2_S$ are similar, and 10-20-fold higher than TER2 [16]. However, in response to DSBs, TER2 is rapidly induced and becomes the predominant TER isoform. The increase in TER2 is coincident with a reduction in telomerase activity. Indeed telomerase inhibition is dependent on TER2: *ter2* mutants do not down-regulate telomerase in response to DNA damage [16]. Telomerase repression is not elicited by replication stress or telomere dysfunction, indicating that TER2-mediated telomerase regulation is specific for DSBs and thus may play a role in repressing DNTF. While the mechanism of TER2-mediated telomerase inhibition is not known, TERT has a higher affinity for TER2 than for TER1 or $TER2_S$, and preferentially assembles into TER2 containing RNP complexes *in vivo*. Therefore, TER2 may serve as a molecular sponge to sequester TERT in a non-functional RNP in response to DSBs [16].

TER is evolving rapidly in Arabidopsis and its relatives. Analysis of sixteen closely related species within the Brassicaceae lineage revealed that these species contain a single locus that bears similarity to the 3' end of TER1 and the 5' end of TER2 from *A*. *thaliana* [27]. Remarkably, several of these TER-like loci lack a template domain altogether, indicating that a functional TER must be encoded elsewhere in the genome. The intervening sequence associated with *A*. *thaliana* TER2 is missing from the TER-like genes of other Brassicaceae. Thus, the appearance of TER2 and its intervening sequence represent recent events likely generated during a massive genome rearrangement that occurred on the branch leading to *A*. *thaliana* [28].

In this study we employ a comparative genomics approach to investigate the regulatory function of TER2. Using data acquired from the 1,001 Arabidopsis genomes project, we show that the intervening sequence in TER2 has the characteristics of a solo long terminal repeat (LTR) from a Copia-like retrotransposon. The element is associated with most, but not all of the TER2 loci. We report that the unique regulatory functions of TER2, including its responsiveness to DSBs, are derived from this transposable element. Consequently, invasion of the TER2 locus by a transposon transformed this lncRNA into a highly sensitive DNA damage sensor that modulates telomerase enzyme activity.

Results

The intervening sequence within TER2 is retained in most but not all *A. thaliana* accessions

Since a clear TER2 ortholog could not be discerned in other members of the Brassicaceae, we analyzed genomic sequence data for different *A. thaliana* accessions, natural strains of *A. thaliana* collected from the wild. *A. thaliana* diverged from its closest relative 10 million years ago [29]. It is estimated that Col-0 and Ler-0, the two best studied *A. thaliana* accessions, are approximately 200,000 years divergent from one another [30]. We retrieved TER1 and TER2 loci from 853 accessions compiled by the 1001 Arabidopsis genomes project (http://signal.salk.edu/atg1001) and analyzed them for variation against Col-0, the *A. thaliana* reference genome where a regulatory function for TER2 was first described [16]. The *TER1* locus is highly conserved, including the 5' and 3' regions flanking CR1 and CR2 (Fig 1A), which lie upstream of the *RAD52* coding region or within a predicted intron [27,31]. *TER1* exhibits 92% identity across the sequenced accessions, but a few polymorphisms are scattered across the RNA (Fig 1A and 1B, S1A Fig). The most notable variations lie within the TER1 template domain (S2A Fig). A transition of A to C occurred three times while a T-A transversion appeared in 44/853 accessions. In neither instance are the two variations found within the same *TER1* gene. Because the *A. thaliana* TER template is 11 nt in length and encodes one and a half copies of the telomere repeat, these TER1 RNAs retain the potential to direct synthesis of TTTAGGG repeats. More intriguing is the C to T mutation in the middle of the template in Bela-1 (S2A Fig). Whether this variation reflects a sequencing error or indicates that an alternative TER1 locus is present in this accession is unknown.

Like TER1 much of TER2 is strongly conserved. CR1 retains high percent identity among the accessions (92%) (Fig 1C). CR2 and the 3'R are also very well conserved with complete conservation in >60% of the accessions analyzed (S3 Fig). Conservation of 3'R was unanticipated since this segment of TER2 is eliminated in the production of $TER2_S$ (Fig 1A). Nevertheless, the high degree of conservation in CR1, CR2 and 3'R argues that these regions are important for TER2 function.

Although the intervening sequence within TER2 is completely conserved in more than 60% of the accessions, striking sequence divergence was observed in many of the other accessions. Two islands of conservation with $\geq$ 50% identity were identified within the intervening sequence, one corresponding to 63 nt and a second of 123 nt (S2B Fig). Hyper-variable sequences flank these regions within the 65 accessions bearing an incomplete intervening sequence. To verify the TER2 sequencing data, we performed PCR genotyping on a sampling of accessions predicted to harbor an intact intervening sequence (Col-0, Ws-2), a partial intervening sequence (Aa-0, Ang-0, Co-1 and Ei-2) or no intervening sequence (Ler-0). PCR primers were positioned within CR1 and 3'R (S4A Fig). A 784 bp PCR product is expected for accessions bearing an intact intervening sequence, a 255 nt product for accessions completely lacking the intervening sequence, and an intermediate size product for accessions with a

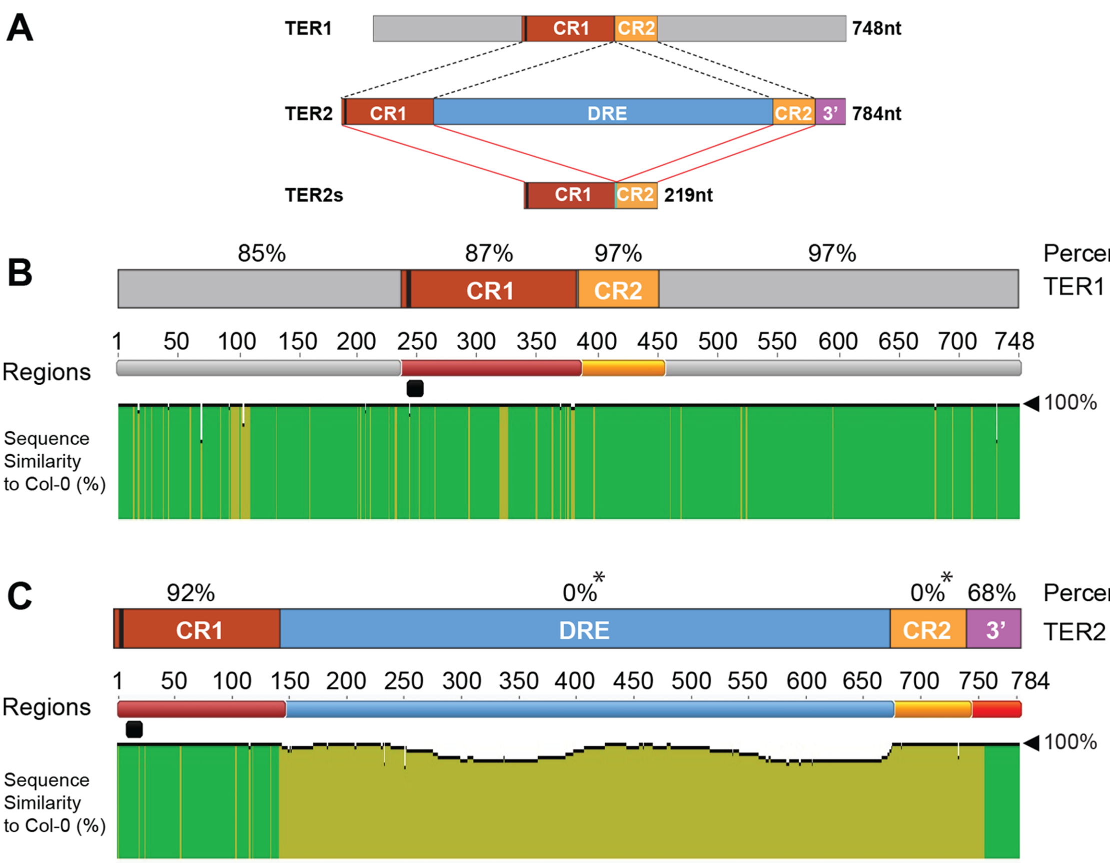

Fig 1. Analysis of TER1 and TER2 loci across *A. thaliana* accessions. (A) Schematic diagram of TER1, TER2, and $TER2_S$. TER1 and TER2 share a core region of ~219 nt comprised of conserved regions 1 and 2 (CR1 and CR2). The telomere template is denoted by a vertical black bar in CR1. $TER2_S$ is formed by splicing to remove the DSB responsive element (DRE) and elimination of the 3' terminus (3' R). (B) Analysis of TER1 among 853 *A. thaliana* accessions. Identity shown in green denotes regions 100% nucleotide similarity whereas mustard yellow indicates variation. There is one colored line for each nucleotide. The height of the bar indicates the degree of variation. Percent identity for each region is denoted in % above each RNA region or for the entire RNA to the right. The telomere template region is indicated by the horizontal black bar. (C) Analysis of TER2 in 853 accessions. Color scheme is the same as in (B). Asterisk indicates that for percent identity to be calculated in a given region, sequence data must be present in all accessions. Sequence was missing for DRE and CR2 for some accessions, and hence these regions are listed as having 0% ID. However, >60% of the accessions were 100% conserved in DRE, and 98% of accessions were 99% conserved in CR2. See S2 Fig for complete alignment of TER2 in 853 accessions.

doi:10.1371/journal.pgen.1005281.g001

partial intervening sequence. Products of the expected sizes were obtained for loci predicted to contain an intact or no intervening sequence, but for all TER2 loci predicted to contain a partial intervening sequence, the genotyping results indicated that this element was completely absent (S4B Fig). Genotyping repeated with siblings from accessions predicted to contain a partial intervening sequence gave the same result (S4C Fig). Genotyping was performed on several additional accessions reported to contain a partial intervening sequence (S1 Table). In all cases, the intervening sequence was absent. Finally, PCR products were sequenced from TER1 and TER2 reactions, with TER1 polymorphisms serving as a control to ensure that seed stocks were as expected (S4B and S4D Fig). The sequencing results confirmed the PCR genotyping data. For all partial intervening sequence accessions tested, there was complete loss of this element. The sequencing data also revealed a substantial deletion (~20 bp) within CR2 in two accessions (S4D Fig).

The simplest explanation for these genotyping results is that the TER2 locus was misannotated in some of the *A. thaliana* accessions. However, we cannot exclude the possibility that the intervening sequence within TER2 is extremely labile and between the time the

genome sequencing was performed and our acquisition of seeds, partially deleted elements were completely eliminated.

The intervening sequence within TER2 is derived from a Copia-like solo LTR

For reasons discussed below, we named the intervening sequence within TER2 DSB responsive element (DRE). BLAST analyses against the *A*. *thaliana* genome using DRE as a query returned two hits, one on the left arm of chromosome 3 (adjacent to At3G30120) bearing 94.6% identity to DRE_{TER2} termed DRE_{3L}, and another on the right arm of chromosome 3 (adjacent to At3G50120) showing 63.4% identity called DRE_{3R} (Fig 2A). Both DRE_{3L} and DRE_{3R} are found within intergenic regions and display a number of single-nucleotide polymorphisms among *A*. *thaliana* accessions (S5A Fig).

BLAST was performed to determine if the DRE is present in other species within the Brassicaceae family. *Arabidopsis lyrata*, *A*. *thaliana's* closest relative, contains 32 copies of DRE dispersed throughout the genome (Fig 2B). A significant fraction of these elements exhibit a high degree of similarity within the 5' 200nt of DRE_{TER2}, and are associated with open reading frames encoding typical retrotransposon proteins (S6 Fig). Three DREs were also detected in *Capsella rubella*, four in *Brassica rapa*, and ten in *Eutrema salsugineum* (Fig 2B). The presence of multiple copies of DRE in *A*. *thaliana* and its relatives suggests that it is a transposable element (TE). Consistent with this conclusion, sequences at the 5' and 3' borders of DRE_{TER2} contain a 5 nt tandem inverted repeat of TGTTG/ACAAC (Fig 2C, brown bar). The tandem inverted repeat at the 5' and 3' boundaries of DRE_{TER2} and DRE_{3L} are highly conserved across the *A*. *thaliana* accessions and are present at the boundaries of DREs detected in other species (S6 Fig). In addition, a target site duplication of TCGTC is present at the 3' end of CR1 and the 5' end of CR2 of TER2 (Fig 2C, green bar). Tandem site duplications flank all three DREs in *A*. *thaliana*, ranging in length from 5 nt for DRE_{TER2} and DRE_{3L} to 18nt for DRE_{3R} (Fig 2C, green bar). The tandem site duplication sequence varies, consistent with the hypothesis that these insertions represent unique TE insertion events rather than gene duplications. The small size of DRE and its association with tandem inverted repeats and target site duplications suggest that DRE is derived from a solo LTR of the abundant Copia family. Based on synteny mapping with *Arabidopsis lyrata* we confirmed that all three Copia-like solo LTRs in *A*. *thaliana* ($TER2_{DRE}$, DRE_{3L}, and DRE_{3R}) are unique insertion events and are of approximately the same age (S7 and S8 Figs). Since the large majority of *A*. *thaliana* accessions apparently harbor an intact DRE within the TER2 locus, it is likely that the element was inserted soon after the TER duplication and was subsequently lost in a small subset of accessions.

Differential expression of TER2 and TER2Δ

The presence of two distinct TER2 alleles in *A*. *thaliana* provided us with an opportunity to study the functional impact of DRE. We previously showed that two RNA transcripts are derived from the Col-0 TER2 locus: the primary TER2 transcript and a processed isoform, $TER2_S$, in which DRE_{TER2} is removed along with 3'R [16,26]. In the Ler-0 accession, the TER2 locus lacks DRE, and thus the primary transcript is predicted to be TER2Δ. To assay for TER2Δ, RT-PCR was performed on RNA from Ler-0 seedlings using primers directed at CR1 and 3'R, which is unique to TER2 (Fig 3A and 3B). A product of the expected size was generated, indicating that a Ler-0 transcript containing CR1, CR2 and 3'R is present. Sequence analysis confirmed this conclusion. Notably, the CR1/CR2 junction in Ler-0 TER2Δ is distinct from Col-0 $TER2_S$ [26] as it contains only a single 5' TCGTC 3' motif instead of the two found in Col-0 (Fig 3B bottom, underlined sequence). Although a faint signal for TER2 was observed

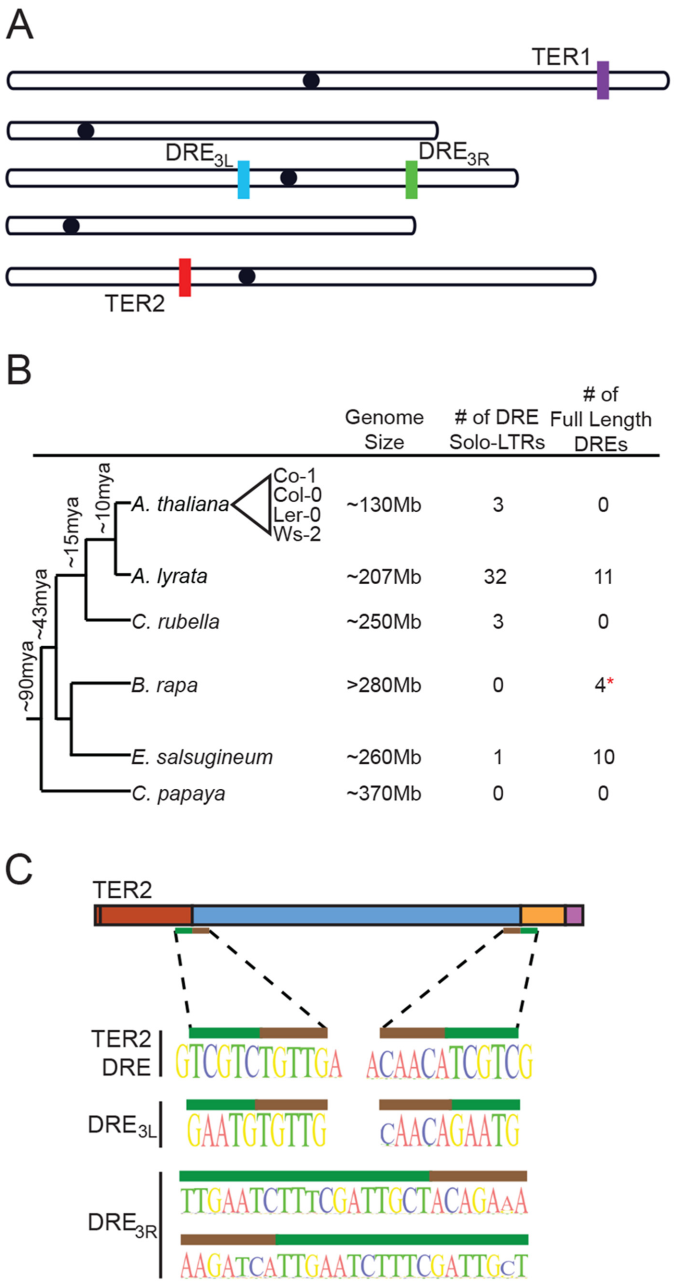

Fig 2. The TER2 intervening sequence has the properties of a Copia-like solo LTR. (A) Schematic of the five chromosomes in *A. thaliana* Col-0 illustrating the locations of TER1, TER2 and DRE on the left arm of chromosome 3 (DRE_{3L}) and the right arm of chromosome 3 (DRE_{3R}) (schematic adapted from TAIR). (B) Phylogenetic tree of select Brassicaceae members (including the Brassicales member *Carica papaya*). The number of solo and full-length DREs identified by BLAST are shown to the right. Approximate time of divergence was adapted from [29]. Representative *A. thaliana* accessions re indicated by the triangle. (C) Sequences at the 5' and 3' boundary elements of DRE in TER2 (top), DRE_{3L} (middle), and DRE_{3R} (bottom) are shown. Nucleotides within the target site duplication are denoted by the green bar and tandem inverted repeats of DRE are represented by the brown bar.

doi:10.1371/journal.pgen.1005281.g002

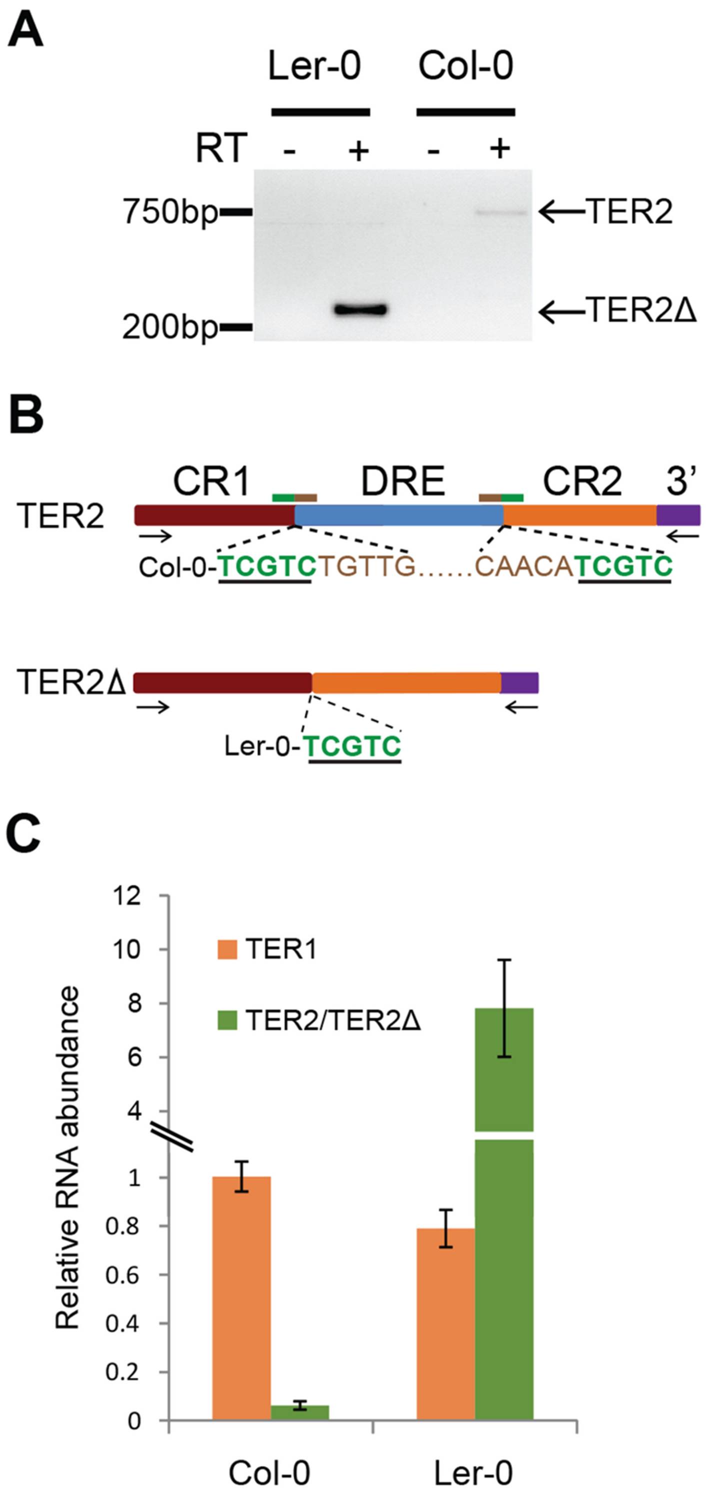

Fig 3. Expression of TER2Δ and association with TERT. (A) RT-PCR results for TER2Δ in Ler-0 and TER2 in Col-0. Primer positions are indicated by arrows in panel B. (B) Schematic showing sequencing results for TER2 and TER2Δ PCR products from Col-0 and Ler-0 obtained from (A). The target site duplication is indicated by the green underlined nucleotides. Tandem inverted repeats are indicated by brown nucleotides. (C) qPCR results for TER1, TER2 and TER2Δ in Col-0 and Ler-0. For comparison, the Col-0 TER1 level was set to 1.

doi:10.1371/journal.pgen.1005281.g003

in Col-0 using our PCR conditions, TER2Δ was not ([Fig 3A](#)), suggesting that TER2Δ is either a transient processing intermediate, or is not generated during the conversion of TER2 to $TER2_S$.

Col-0 TER2 is a poorly expressed transcript (Fig 3A) and is substantially less abundant than TER1 or $TER2_S$ [16]. To assess the relative abundance of Ler-0 TER2Δ, we performed qPCR (Fig 3C). The steady state level of TER1 was similar in Ler-0 and Col-0. However, Ler-0 TER2Δ was approximately 6–8 fold more abundant than Ler-0 TER1. By comparison, Col-0 TER2 was 15–20 fold less abundant than Col-0 TER1 (Fig 3C). Thus, Col-0 TER2 and Ler-0 TER2Δ are differentially regulated *in vivo*.

In Col-0, TER2 but not TER1 or $TER2_S$ is rapidly induced by DSBs [16]. Therefore, we asked if regulation is confined to TER2 by examining TER2 and TER2Δ in other *A*. *thaliana* accessions (Fig 4A). Seven day-old Ler-0 and Col-0 seedlings were treated with 20μM zeocin and qPCR was performed. In control reactions, BRCA1 mRNA was induced in both accessions after 2 hours and peaked at 4 hours, confirming that a DNA damage response was elicited (Fig 4B). As expected, the level of TER1 was unchanged in Ler-0 and Col-0 following zeocin treatment (S9 Fig). In addition, Col-0 TER2 increased 2.5 fold after 2 hours in zeocin relative to untreated seedlings (Fig 4C). In marked contrast, there was no significant change in TER2Δ over the 4 hour zeocin treatment (Fig 4C).

To test if DSB-mediated regulation of TER2 is a peculiarity of the Col-0 accession, we examined TER2/TER2Δ transcripts in two additional accessions: Ws-2, which contains DRE_{TER2} and Co-1, which lacks it (Fig 4D). Consistent with the findings in Ler-0 and Col-0, there was no change in Co-1 TER2Δ, while Ws-2 TER2 was induced (Fig 4D). We conclude that the effect of DSBs on TER2 varies across *A*. *thaliana* accessions, and correlates with the presence of DRE_{TER2}.

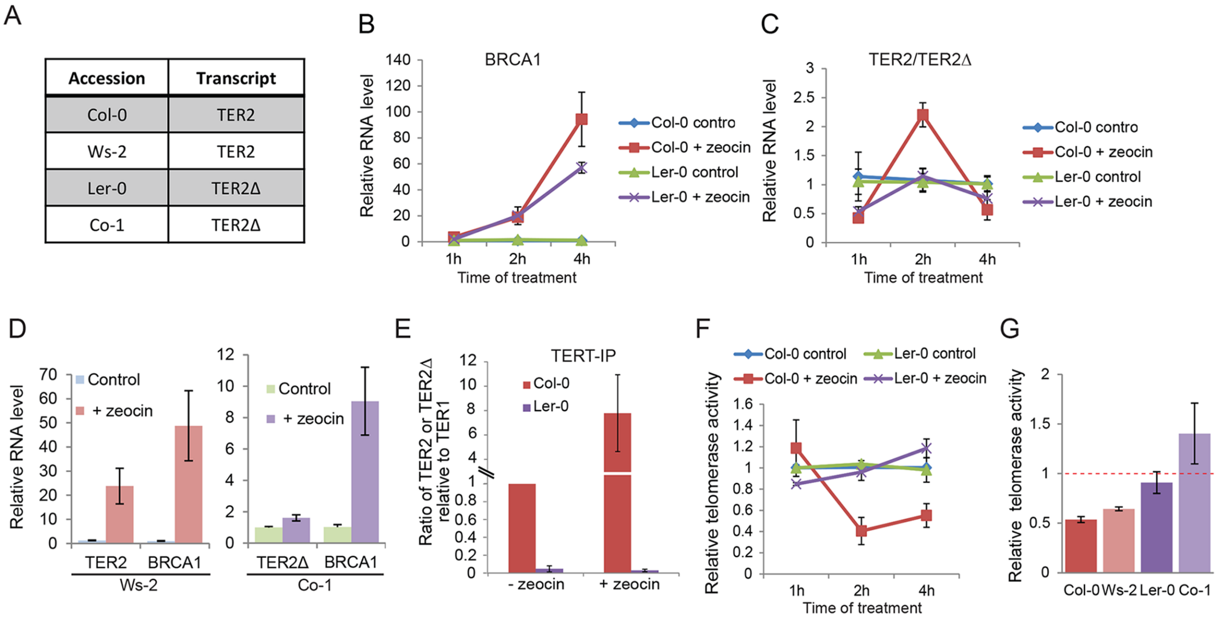

Fig 4. DSB-mediated RNA induction and telomerase inhibition are associated with DRE. (A) Table indicating the TER2 transcript status for four *A*. *thaliana* accessions. (B) and (C) show qPCR results for Col-0 and Ler-0 seedlings treated with zeocin for the time points indicated. Data for the BRCA1 control (B) and TER2 (Col-0) or TER2Δ (Ler-0) transcripts (C) are shown. (D) qPCR results for accessions with TER2 (Ws-2) and TER2Δ (Co-1) submitted to the zeocin regimen for 2 h. (E) qPCR results following TERT immunoprecipitation in Col-0 and Ler-0 seedlings treated with or without zeocin (time point). The TER2:TER1 ratio in Col-0 and the TER2Δ:TER1 ratio in Ler-0 are shown. Values were normalized to Col-0 TER2:TER1 ratio in the absence of zeocin (set to 1). (F) qTRAP results for Col-0 and Ler-0 seedlings with or without zeocin treatment. (G) qTRAP results for the samples in (D) and the 2 h time point from (C). Telomerase activity was normalized to the corresponding untreated controls and set to 1. Red dashed bar indicates no change between treated and untreated samples. The changes in telomerase activity in Col-0 and Ws-2 were statistically significant (p-value< 0.05). Significance was calculated relative to untreated samples using a Student's *t*-test. For all experiments, n > 3.

doi:10.1371/journal.pgen.1005281.g004

We next asked if transcripts were derived from the other two DRE-like sequences in Col-0, and if so whether they responded to DSBs. Semi-quantitative RT-PCR was performed with primers specific for DRE_{3L} and DRE_{3R} on seedlings in the presence or absence of zeocin (S5B Fig). DRE_{3L} transcripts could not be detected under either condition. However, transcripts from DRE_{3R} were observed in the presence of zeocin (S5B Fig), indicating that a DNA damage-sensing element resides within DRE_{TER2} as well as DRE_{3R}

TERT preferentially associates with TER2 over TER2Δ *in vivo*

We previously showed that Col-0 TERT displays a hierarchy of binding favoring TER2 > TER1 >> $TER2_S$ both *in vitro* and *in vivo* [16]. The molecular basis for the enhanced affinity of TERT for TER2 is known. Since DRE_{TER2} and the 3'R are unique to TER2, it seems likely that one of these elements influences TERT binding. To investigate this possibility, we examined the relative affinity of TERT for TER2Δ. Col-0 and Ler-0 seedlings were subjected to immunoprecipitation with TERT antibody followed by qPCR (Fig 4E). We set the ratio of TER2 to TER1 in the Col-0 TERT IP to 1, and then assessed the change in TERT-bound TER2 following zeocin treatment. The relative abundance of TER2 containing TERT complexes increased ~ 7-fold in response to DSBs (Fig 4E). Since the input level of TER2 increased by only 2.5-fold under these conditions (Fig 4C), the data raise the interesting possibility that other DNA damage-induced factors promote TER2 assembly with TERT. In marked contrast to TER2, we found that TER2Δ is not a preferred binding partner for TERT *in vivo*, and further zeocin treatment did not change the relative abundance of TER2Δ containing TERT complexes (Fig 4E). These results argue that the increased affinity of TERT for TER2 in Col-0 reflects the presence of DRE_{TER2} and not 3'R.

Accessions lacking DRE_{TER2} do not exhibit DSB-induced telomerase inhibition

Since Col-0 plants lacking TER2 do not down-regulate telomerase activity in response to DSBs [16], we asked if DSB-induced telomerase regulation is dependent on DRE_{TER2} by comparing the level of telomerase activity in Ler-0 and Col-0 in the presence of zeocin. As expected application of quantitative telomere repeat amplification protocol (qTRAP) to Col-0 seedlings treated with zeocin for 2 or 3 hours showed reduced telomerase activity (70% decrease) compared to untreated seedlings (Fig 4F and S10 Fig). Although there was an alleviation of the inhibitory effect after 3–4 hours of treatment, enzyme activity was maintained at 50% of untreated level. In contrast, under the same treatment regime, telomerase activity was unaltered in Ler-0 (Fig 4F). Similar results were obtained with Ws-2 (plus DRE_{TER2}) and Co-1 (minus DRE_{TER2}) accessions, respectively (Fig 4G). These findings imply that DRE is necessary for DSB-induced telomerase repression.

To further assess the role of DRE in telomerase regulation, we generated two transgenic *A. thaliana* lines. First we asked if the presence of TER2 was sufficient to alter the level of telomerase activity in Ler-0 by expressing TER2 from its native promoter in this accession. In one of the transformants, the steady state level of transgenic TER2 was higher (2.5 fold) than the basal level of endogenous TER2 in wild type Col-0 (Fig 5A). qTRAP revealed a small, but statistically significant decrease in telomerase activity in the transformant (Fig 5B), indicating that Ler-0 telomerase can be down-regulated by Col-0 TER2. Next we asked if over-expression of TER2Δ altered telomerase activity in Col-0. TER2Δ expression was driven by the powerful CaMV promoter in wild type Col-0. As expected, there were no change in TER1 or TER2, but the steady state level of transgenic TER2Δ was ~8-fold higher than endogenous TER2Δ in wild type Ler-0. However, qTRAP showed no change in telomerase activity relative to untransformed Col-0 controls (Fig 5A and 5B). We conclude that the regulation of telomerase by TER2 is dependent on DRE_{TER2}.

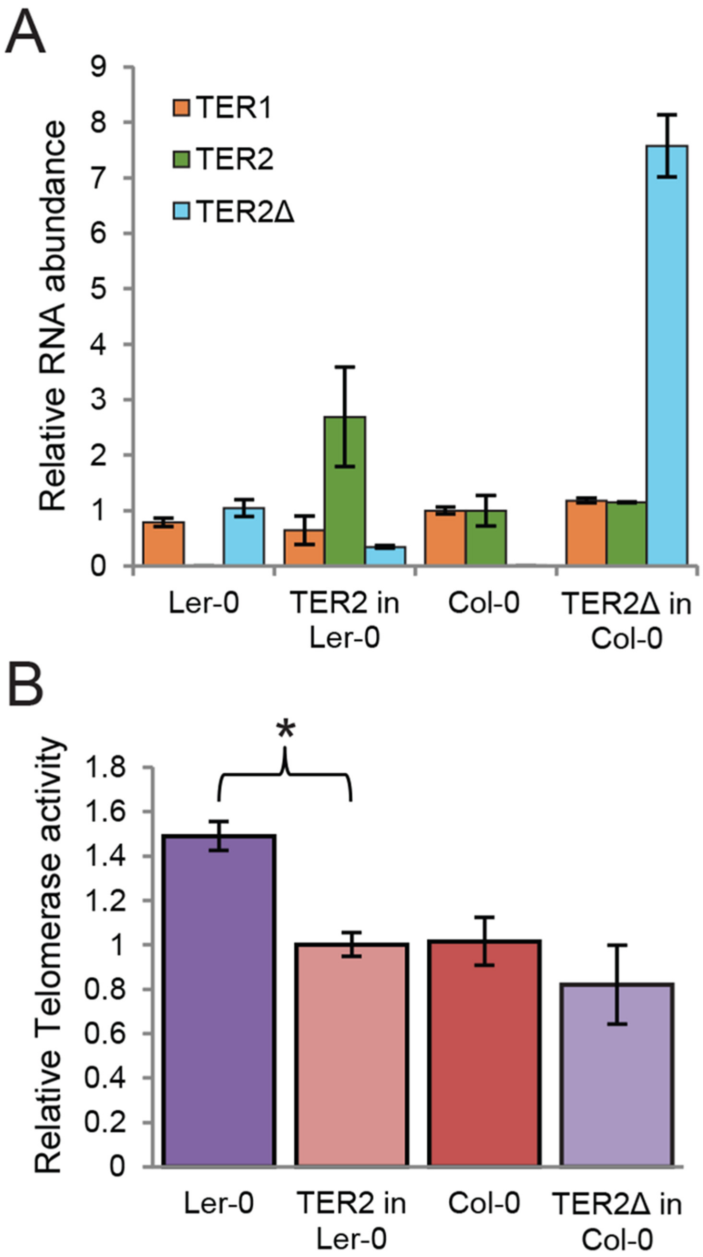

Fig 5. TER2 not TER2Δ represses telomerase activity. (A) qPCR results are shown for transgenic seedlings expressing TER2 in Ler-0 or TER2Δ in Col-0. TER1 and TER2 levels were normalized to the values in wild type Col-0 (set to 1). TER2Δ was normalized to the value in wild type Ler-0 (set to 1). GAPDH served as a reference gene. (B) qTRAP results are shown for the seedlings analyzed in (A). Relative telomerase activity was normalized to wild type Col-0. The change in telomerase activity in Ler-0 transformants expressing TER2 relative to wild type Ler-0 is statistically significant (p-value<0.005). Significance was calculated relative to untreated samples using a Student's *t*-test. For all experiments, n > 3.

doi:10.1371/journal.pgen.1005281.g005

TER2 is an unstable RNA stabilized in response to DSBs

The rapid induction of Col-0 TER2 in response to DSBs could occur through increased TER2 transcription or increased RNA stability. Because the sequences upstream of all *TER2* genes are highly conserved, we considered the former possibility less likely. Indeed, when TER2

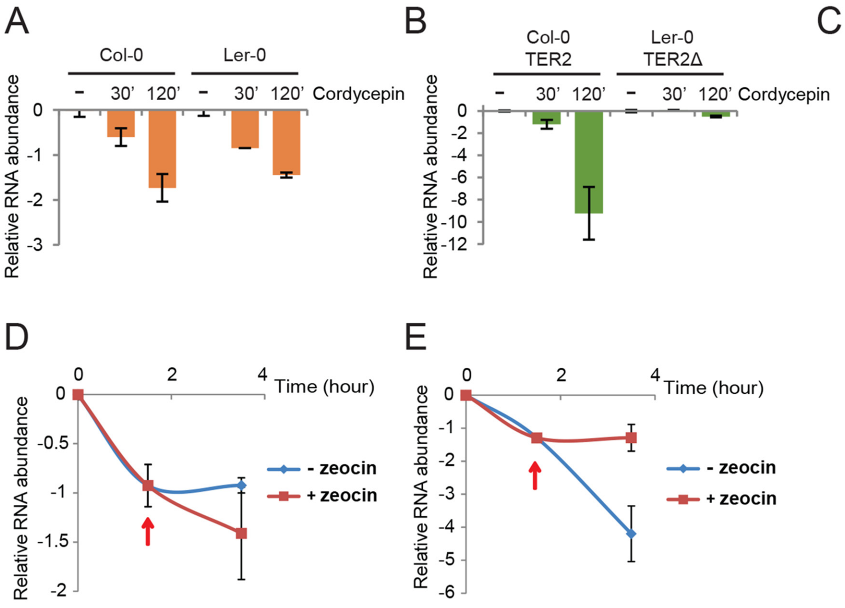

Fig 6. TER2 is a labile RNA transcript stabilized by DNA damage. qPCR results are shown for TER1 and TER2/TER2Δ from Col-0 and Ler-0 in the presence of cordycepin. Col-0 and Ler-0 seedlings were treated with cordycepin (100μg/μl) for the times indicated followed by qPCR to monitor TER1 (A) and TER2/ TER2Δ (B). The values obtained for untreated RNA samples were set to 0 and the fold decrease is shown. eIF-4a was used as reference gene for normalization. (C-E) qPCR results from a time course experiment of Col-0 seedlings treated with cordycepin followed by zeocin. Seedlings were incubated with cordycepin for 1.5 h to shut down transcription, and zeocin was added (red arrows). The incubation continued for a total of 3.5 h. Results for BRCA1 (C), TER1 (D), TER2 (E) are shown.

doi:10.1371/journal.pgen.1005281.g006

transcription was monitored in seedlings expressing a fused GUS reporter to a TER2 or TER2Δ promoter in Col-0 and Ler-0, respectively, approximately the same level of GUS staining was observed in the presence or absence of zeocin (S11 Fig). Hence, TER2 induction in response to DNA damage is not caused by increased transcription.

We assessed TER2 stability using six day-old seedlings treated with the transcription elongation inhibitor cordycepin. TER1 and TER2 RNA levels assessed by qPCR showed that Col-0 and Ler-0 TER1 have similar half-lives, $t_{1/2}$ = 75 and 84 min, respectively (Fig 6A). The stability of TER2Δ was even greater with $t_{1/2}$ = 244 min (Fig 6B). TER2, on the other hand, had a much shorter half-life than either TER2Δ or TER1: TER2 $t_{1/2}$ = 13 min (Fig 6B). Thus, TER2 is an intrinsically unstable transcript.

To test if DSBs reduce TER2 turnover, Col-0 seedlings were treated with cordycepin to pause transcription and then zeocin was added after 90 min to produce DSBs. Although there was a slight change in the abundance of TER1 and BRCA1 mRNA in the presence of zeocin, this change was not statistically significant (Fig 6C and 6D). In contrast, TER2 abundance declined sharply over the 3.5 hour time course, but immediately after the introduction of zeocin, TER2 was stabilized (Fig 6E). These data implicate DRE_{TER2} as the causal factor in destabilizing TER2 and in turn negatively regulating telomerase activity during bouts of DNA damage.

Discussion

When the insertion of a TE within or adjacent to a gene leads to a change in gene function the process is termed "exaptation" [32]. Exaptation can alter gene regulation through myriad different mechanisms. A prominent example in plants is the insertion of multiple TEs adjacent to *teosinte branched1* (tb1), which gave rise to domesticated maize [33]. One of the TEs disrupts a

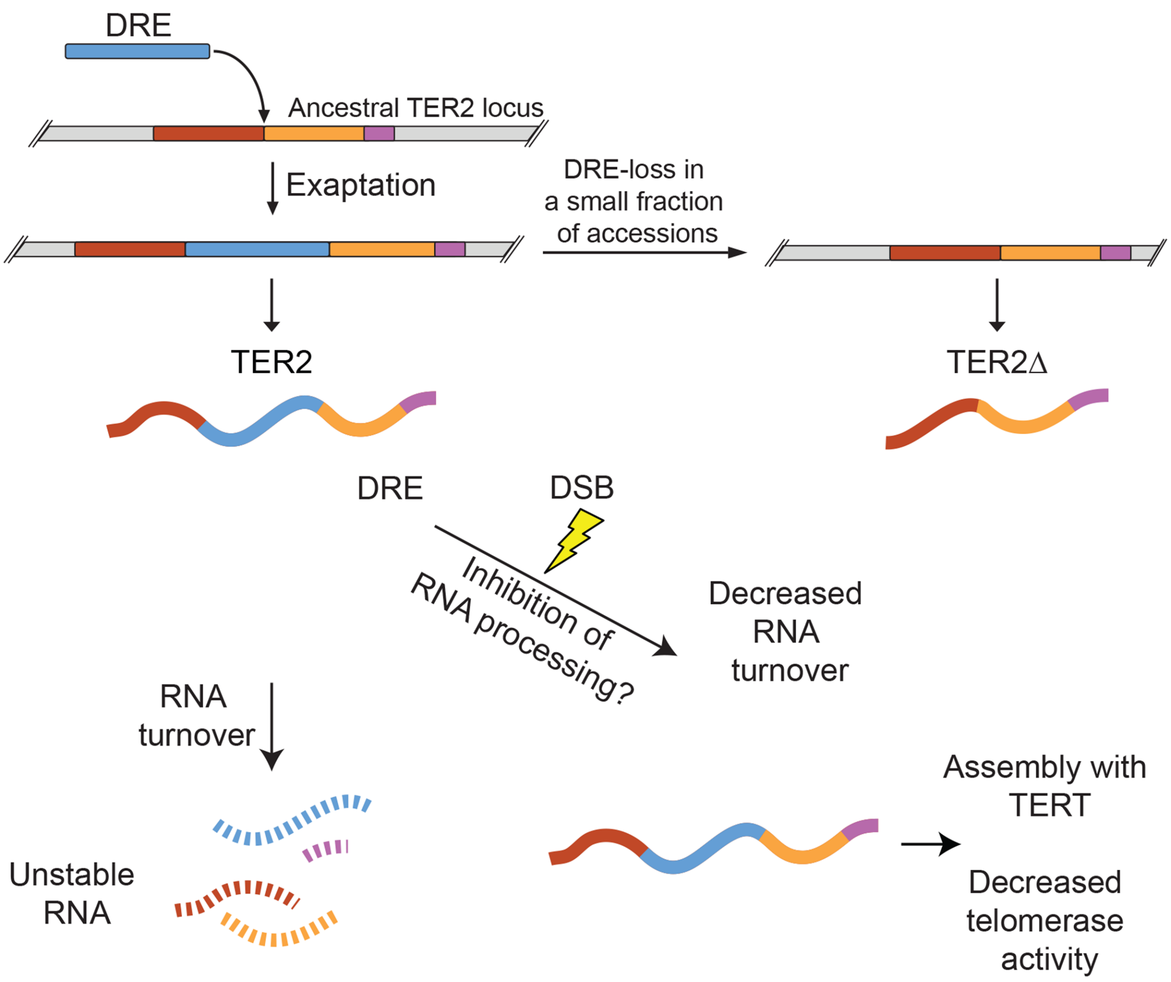

Fig 7. Model for exaptation of a TE into TER2 and the emergence of a telomerase regulatory lncRNA. Duplication of the single copy ancestral TER gene was followed soon thereafter by exaptation of DRE into the *A*. *thaliana* TER2 locus. The majority of *A*. *thaliana* accessions retain DRE (e.g. Col-0), but a small subset lost it (e.g. Ler-0). TER2Δ is produced by accessions lacking DRE. DRE acts as a post-transcriptional sensor that modulates TER2 abundance in response to DNA damage. Under normal physiological conditions, TER2 is an unstable RNA. However, in the presence of DSBs, TER2 is rapidly induced. Whether this is due to direct RNA stabilization or inhibition of TER2 processing (to yield TER2s) is unknown. TER2 has a higher affinity for TERT than TER1 or TER2Δ and following induction by DNA damage accumulates in TERT containing complexes *in vivo*. TER2-mediated telomerase inhibition may reflect competitive inhibition of TER1 for TERT. The transient decrease in telomerase activity may promote DSB repair rather than de novo telomere formation, thereby stabilizing the genome.

doi:10.1371/journal.pgen.1005281.g007

regulatory region of tb1, leading to increased expression and enhanced apical dominance. In vertebrates, exaptation of TEs is more prevalent at lncRNA loci than in protein-coding genes [34]. Approximately 41% of vertebrate lncRNA sequence is derived from TEs [35,36], leading Johnson and Guigo to propose that TEs can behave as pre-formed functional RNA domains, and further that exaptation of TEs is a major driving force in lncRNA evolution [36]. A recent systematic survey in vertebrates catalogued multiple instances of TEs altering lncRNA promoters, splice sites, and polyadenylation sites [37]. LncRNAs can also acquire novel interaction partners as a direct result of TE exaptation [32]. For instance, TEs within XIST facilitate interaction with a host of protein complexes including PRC2 and splicing factor ASF2 [38].

Here we show that invasion of a small TE (DRE) into the *A*. *thaliana* TER2 locus profoundly altered the function of this lncRNA (Fig 7). This exaptation event is not fixed, as the

TER2 genes in 9% of the 853 accessions examined lack DRE. Insertion and subsequent loss of TEs is not uncommon in Arabidopsis. Some 80% of the annotated TEs in *A*. *thaliana* were lost in one or more accessions [39]. In the 200,000 years since Col-0 and Ler-0 diverged, at least 200 TEs have been active, and the unique insertions/deletions between the two accessions have biological implications [30]. One illustrative example of TE exaptation occurred at the Flowering Locus C (FLC) in Ler-0. Insertion of a Mutator-like transposon in this accession decreased FLC transcription, causing early flowering [40]. In this study we exploited the natural genetic heterogeneity within the TER2 locus, and discovered that many of the unique functions ascribed to this lncRNA derive from DRE.

First, DRE destabilizes TER2. A survey of ~800 lncRNAs in mouse revealed that only a small fraction are unstable, defined as RNAs with a half-life of less than 60 minutes [41]. By this criterion, TER2 is a highly unstable transcript with a half-life of only 13 minutes (Fig 7). TER1 ($t_{1/2}$ = 80 min) and TER2Δ ($t_{1/2}$ = 240 min), on the other hand, are categorized as stable RNAs. Unstable lncRNAs, like their unstable mRNA counterparts, are typically associated with regulatory functions, while stable RNAs are thought to serve housekeeping roles [42]. With Col-0 *A*. *thaliana* TER1 and TER2, this paradigm also holds.

A second key observation is that the instability of TER2 arising from DRE is reversed in response to DNA damage (Fig 7). The abundance of TER2, but not TER1 or TER2Δ is elevated in response to DSBs, and this change is largely, if not entirely, dependent on RNA stabilization rather than new transcription. Exaptation of a TE is known to endow host genes with the capacity to respond to environmental cues. For example, a cold-sensitive TE was inserted into the promoter of Ruby, a transcription factor that regulates flesh color in *Citrus sinensis* (blood orange). Cold activates the transposon, which in turn activates Ruby and downstream anthocyanin production [43]. In the case of TER2, DRE imparts DNA damage sensitivity, which increases TER2 abundance. How TER2 is regulated in response to DSBs is unknown. One possibility is that DRE carries binding sites for one or more interaction partners responsive to DNA damage, which then stabilize TER2. RNA binding proteins can play a significant role in the DNA damage response by regulating specific target genes post-transcriptionally [44]. TER2 turnover might be controlled through the small RNA regulatory pathway. A 24 nt RNA is associated with DRE_{TER2} [45]. This finding is particularly intriguing given the recent discovery that small RNAs modulate the response to DSBs in both vertebrates and Arabidopsis [46]. Finally, it is possible that DNA damage blocks the RNA processing steps (e.g. splicing) that lead to production of $TER2_S$ (Fig 7). Splicing machinery has emerged as a target of the DDR [47].

The third key observation from this work is that DRE increases the affinity of TER2 for TERT (Fig 7), and correlates with the down-regulation of telomerase activity. DRE could modify TER2 structure in a manner that enhances its inherit affinity for TERT. Alternatively, DRE may make independent contacts with TERT to increase TER2 affinity. Intriguingly, zeocin treatment causes an even greater enrichment of TER2 containing TERT complexes than expected based on the fold induction of TER2, suggesting that a TER2 associated factor that is also responsive to DNA damage might drive the assembly of TER2-TERT RNPs.

Altogether, our data are consistent with a model in which exaptation of a TE into the *A*. *thaliana* TER2 locus gave rise to a new mode of telomerase regulation. Specifically, we propose that the DRE converted TER2 into a DNA damage sensor that controls telomerase enzyme activity through sequestration of TERT. Furthermore, because this regulatory pathway is regulated by changes in RNA stability, it is both rapidly responsive and reversible, allowing the *A*. *thaliana* accessions that carry DRE to fine-tune telomerase activity when the plant is under genome assault. These discoveries provide a fresh perspective on the role of TE exaptation in shaping lncRNA function and evolution.

Materials and Methods

Plant material, growth conditions and transformation

For experiments with seedlings, seeds from different accessions (Col-0, Ler-0, Ws-2, etc) were sterilized in 50% bleach with 0.1% Triton X-100 and then stored in 4°C for 2–4 days. Liquid Murashige and Skoog (MS) medium were used for germination and growing [16]. After transferring cold-treated seeds to MS, plants were grown at 22°C under long day light condition for ~7 days. The Col-0 TER2 gene including 3kb upstream sequence and 300bp downstream sequence was cloned in the pMDC99 vector for transformation in the Ler-0 background. Hygromycin MS plates were used for selection. For Col-0 transformation, TER2Δ together with 300 bp downstream flanking region was cloned into the pBA002 vector with 35S promoter. BASTA MS plates were used for the selection.

Sequence acquisition and analysis

Sequences corresponding to TER2 (Genbank accession number: HQ401285.1) were obtained using the genome browser at http://signal.salk.edu/atg1001. The search query AT5G24660 was used to pinpoint the region of interest, and all available tracks (accessions) were selected. Two sequences were removed from our analysis. Hov 3–2 was removed because it was the only accession with two deletions in the 5' end, corresponding to 20 nt from the 5' start of TER2, and a 100 nt deletion starting at nucleotide #101. The template region was not disturbed in this accession, possibly indicating a functional TER2 is generated. The Tottarp-2 accession was removed because the sequence corresponding to our search region did not contain sequences corresponding to TER2, most importantly, a template region.

Sequences were trimmed in MEGA5, and then analyzed using Geneious v6.0 (Biomatters). Sequence conservation and alignments were performed using Geneious. DRE-like sequences were obtained by BLAST searches of the *A. thaliana* (www.arabidopsis.org), *A. lyrata*, *Capsella rubella*, *Brassica rapa*, and *Thellungiella halophila* genomes accessed via www.phytozome.net v9.1 [48,49].

DNA damage treatment and assays

A. thaliana seedlings (5–7 day old) were transferred to fresh MS liquid medium with 20 μM zeocin (Invitrogen) as described [16]. Seedlings were kept in the dark with gentle agitation for 1, 2 or 4 h. Multiple seedlings were combined and flash frozen in liquid nitrogen for RNA extraction or protein extraction for TRAP. The combined sample was treated as a single biological replicate.

Nucleic acid extraction, genotyping and PCR

DNA samples were prepared from the leaves of different accessions. Both TER1 and TER2 loci were used for genotyping. PCR samples were resolved in 1% agrose and gel purified and sequenced. RNA was extracted from seedlings using the Direct-zol RNA MiniPrep kit (Zymo Research, Epigenetics) according to the manufacturer's instructions. 1 μg total RNA was used for preparing cDNA. For RT-PCR, cDNA was synthesized by SuperscriptIII Reverse Transcriptase (Invitrogen) using random primers. For qRT-PCR, reverse transcription was performed using the Superscript cDNA master mix (Quanta), according to the manufacturer's instructions. 1:5 diluted cDNA was used for qPCR. qPCR was performed on a Bio-Rad CFX-1000 using the following primers: qTER2Δ F: 5'-AGAACGTTGACGGCTAAAGG-3'; qTER2Δ R: 5'- TGTGGCATAAGGCAAACTGA-3'; TER2, BRCA1, TER1 and GAPDH primers are

used as described before [16]. Data were analyzed using Bio-Rad's CFX manager software. ΔΔCT values were obtained by comparing against GAPDH levels.

qTRAP and immunoprecipitation (IP) qRT-PCR

qTRAP assays were performed as described [50]. Data were normalized against untreated Col-0. For immunoprecipitation, TERT antibody [50] was conjugated with Dynabeads Protein A (Invitrogen) then incubated with protein extracts in 4°C. RNA was recovered from the IP sample using phenol/chloroform followed by ethanol precipitation [16]. qPCR was performed on TER1 and TER2/TER2Δ. The ΔCT value was used to determine the relative level of TER2 or TER2Δ against TER1.

RNA stability assays

5–7 day old seedlings were treated with cordycepin (100 ng/μl as a working concentration) for 2 h before RNA extraction. RNA was analyzed by qPCR normalized to eIF-4a [51]. RNA abundance was converted to the decreased level relative to untreated. RNA half-life was determined by the absolute value of inverse of the slope of the equation plotted by untreated and treated data. For the combined cordycepin/zeocin experiment, seedlings were pre-incubated with cordycepin for 1.5 h followed by zeocin and the incubation was continued for 2 h. RNA extraction and qPCR were used to determine RNA abundance. RNA half-life was determined by plotting RNA abundance versus time as described in [51].

GUS staining

3 kb of sequence upstream of the TER2 5' terminus was cloned in a GUS reporter vector pMDC163. The construct was transformed into *A. thaliana* Col-0 and Ler-0 as described [52]. After selection in hygromycin, transformants seedlings were treated with zeocin for 2 h and then subjected to GUS histochemical staining as described [53].

Supporting Information

S1 Table. Summary of the status of DRE at the TER2 locus in all accessions tested.
(PDF)

S1 Fig. Complete alignment of TER1 from the 853 Arabidopsis accessions. Complete FASTA alignment of TER1 from 508 Arabidopsis accessions. All accessions with sequence data for this locus were included in this alignment. Lack of sequence data for a particular regions is indicated by a (?).
(FASTA)

S2 Fig. Polymorphisms within the TER1 template and the TER2 intervening sequences across accessions. (A) Screenshot of a Geneious alignment of TER1 template regions amongst 853 *A. thaliana* accessions (grey box). Three types of polymorphisms are observed. The arrows point to the nucleotide changes and their observed frequency. (B) Alignment of only the accessions showing partial intron loss within TER2. The green bar indicates complete nucleotide identity among accessions. Height of the yellow bars is indicative of nucleotide variation or loss of nucleotide sequence at that position. Two conserved regions in the intron (DRE) are highlighted by horizontal blue bars. The dashed line denotes two hypervariable regions.
(PDF)

S3 Fig. Complete alignment of TER2 from the 853 Arabidopsis accessions. Complete FASTA alignment of TER2 from 510 Arabidopsis accessions. All accessions with sequence data

for this locus were included in this alignment. Lack of sequence data for a particular regions is indicated by a (?).
(FASTA)

S4 Fig. Genotyping analysis of *A. thaliana* TER2 loci. (A) Schematic map of the status of the TER2 intervening sequence in different accessions. The positions of PCR primers are indicated by black arrows. (B) Genotyping results for TER1 and TER2 loci in different accessions. TER2 PCR products with the full-length DRE are expected to be ~750 bp, and PCR products lacking DRE are ~200 bp. Col-0 was used as a full-length DRE control, and Ler-0 used as complete DRE loss control. Sequence analysis of all of the TER1 PCR products confirmed accession identity. (C) TER2 DRE genotyping results in four siblings of each accession. (D) Sequencing data for TER2 genotyping PCR products in (B). The gaps in DRE and CR2 demonstrate sequence loss for these accessions.
(PDF)

S5 Fig. Sequence conservation of DRE_{3L} and DRE_{3R} among different *A. thaliana* accessions and their expression in response to DNA damage. (A) DRE_{3L} and DRE_{3R} conservation across *A. thaliana* ecotypes. (B) RT-PCR results for DRE_{3L} and DRE_{3R} with or without zeocin treatment. The expected sizes of the PCR products are highlighted by arrows.
(PDF)

S6 Fig. Full length DREs are found throughout Brassicaceae with species-specific family expansions. Schematic representation of Brassicaceae DRE organization. Name and length (in base pairs) are on the right. LTRs (black arrows in blue background) as well as intact Gag, Integrase and RT (Reverse Transcriptase) ORFs are indicated. BrDRE-1 and BrDRE-3 encode for all intact Pol components (Integrase and RT) except for RNase. ThDRE-2 does not contain a second LTR, but shows high sequence similarity to ThDRE-1 throughout. AlDRE-2 does not contain an ORF, and shows low sequence similarity between LTRs.
(PDF)

S7 Fig. Syntenic analysis of DRE_{3R}. CoGe (https://genomevolution.org/CoGe/) screenshot depicting synteny at the locus harboring DRE_{3R} in *A. thaliana* and the closest relative, *A. lyrata*. Purple box indicates where the transposable element is located in *A. thaliana* (bottom genome). The pink rectangles denote regions of synteny between *A. thaliana* and *A. lyrata* (top genome). The gap in synteny between the two species where the transposable element resides in *A. thaliana* is indicative of a recent insertion.
(PDF)

S8 Fig. Syntenic analysis of DRE_{3L}. CoGe screenshot of the *DRE_{3L}* locus. Purple box indicates where the transposable element is located in *A. thaliana* (bottom genome). The pink rectangles denote regions of synteny between *A. thaliana* and *A. lyrata* (top genome). A large section of DNA has been inserted in *A. thaliana* that is not present in *A. lyrata*. In *A. thaliana*, the inserted DNA includes a pseudogene (grey bars) and the DRE_{3L} (purple box). Sequence similar to the region surrounding DRE_{3L} is not found anywhere else in the *A. lyrata* or *A. thaliana* genomes.
(PDF)

S9 Fig. The steady state level of TER1 is unchanged in Col-0 or Ler-0 in response to DSBs. qPCR results for TER1. The reaction was performed in parallel with the experiments shown in Fig 4B and 4C.
(PDF)

S10 Fig. TER2 induction is an early response to DNA damage in Col-0. The experimental design was the same as in Fig 4, with the addition of a 3 hour time point. BRCA1 (A), TER2 (B) and TER1 (C) RNA transcripts were determined by qRT-PCR. Telomerase activity was determined by qTRAP (D). The X-axis indicates time of zeocin treatment
(PDF)

S11 Fig. TER2 promoter activity in Col-0 and Ler-0. Sequences 3kb upstream of TER2 were cloned into a vector containing the GUS gene as a reporter. The construct was transformed into both Col-0 and Ler-0. Seven day-old seedlings were treated with zeocin for 2 hours and then tested for GUS activity.
(PDF)

Acknowledgments

We thank Kara Boltz for preparation of the GUS construct and Jennifer Townley for assistance with RNA extractions.

Author Contributions

Conceived and designed the experiments: HX ADLN DES. Performed the experiments: HX ADLN. Analyzed the data: HX ADLN DES. Contributed reagents/materials/analysis tools: HX ADLN. Wrote the paper: HX ADLN DES.

References

1. Lee JT, Strauss WM, Dausman JA, Jaenisch R (1996) A 450 kb transgene displays properties of the mammalian X-inactivation center. Cell 86: 83–94. PMID: 8689690
2. Guttman M, Donaghey J, Carey BW, Garber M, Grenier JK, et al. (2011) lincRNAs act in the circuitry controlling pluripotency and differentiation. Nature 477: 295–300. doi: 10.1038/nature10398 PMID: 21874018
3. Scheuermann JC, Boyer LA (2013) Getting to the heart of the matter: long non-coding RNAs in cardiac development and disease. EMBO J 32: 1805–1816. doi: 10.1038/emboj.2013.134 PMID: 23756463
4. Ponting CP, Oliver PL, Reik W (2009) Evolution and functions of long noncoding RNAs. Cell 136: 629–641. doi: 10.1016/j.cell.2009.02.006 PMID: 19239885
5. Gunes C, Rudolph KL (2013) The role of telomeres in stem cells and cancer. Cell 152: 390–393. doi: 10.1016/j.cell.2013.01.010 PMID: 23374336
6. Bernardes de Jesus B, Blasco MA (2013) Telomerase at the intersection of cancer and aging. Trends Genet 29: 513–520. doi: 10.1016/j.tig.2013.06.007 PMID: 23876621
7. Cifuentes-Rojas C, Shippen DE (2012) Telomerase regulation. Mutat Res 730: 20–27. doi: 10.1016/j.mrfmmm.2011.10.003 PMID: 22032831
8. Karamysheva Z, Wang L, Shrode T, Bednenko J, Hurley LA, et al. (2003) Developmentally programmed gene elimination in *Euplotes crassus* facilitates a switch in the telomerase catalytic subunit. Cell 113: 565–576. PMID: 12787498
9. Wong MS, Wright WE, Shay JW (2014) Alternative splicing regulation of telomerase: a new paradigm? Trends Genet 30: 430–438. doi: 10.1016/j.tig.2014.07.006 PMID: 25172021
10. McClintock B (1941) The stability of broken ends of chromosomes in *Zea mays*. Genetics 26: 234–282. PMID: 17247004
11. Ribeyre C, Shore D (2013) Regulation of telomere addition at DNA double-strand breaks. Chromosoma 122: 159–173. doi: 10.1007/s00412-013-0404-2 PMID: 23504035
12. Zhang W, Durocher D (2010) De novo telomere formation is suppressed by the Mec1-dependent inhibition of Cdc13 accumulation at DNA breaks. Genes Dev 24: 502–515. doi: 10.1101/gad.1869110 PMID: 20194442
13. Makovets S, Blackburn EH (2009) DNA damage signalling prevents deleterious telomere addition at DNA breaks. Nat Cell Biol 11: 1383–1386. doi: 10.1038/ncb1985 PMID: 19838171
14. Kharbanda S, Kumar V, Dhar S, Pandey P, Chen C, et al. (2000) Regulation of the hTERT telomerase catalytic subunit by the c-Abl tyrosine kinase. Curr Biol 10: 568–575. PMID: 10837221

15. Wong JM, Kusdra L, Collins K (2002) Subnuclear shuttling of human telomerase induced by transformation and DNA damage. Nat Cell Biol 4: 731–736. PMID: 12198499
16. Cifuentes-Rojas C, Nelson AD, Boltz KA, Kannan K, She X, et al. (2012) An alternative telomerase RNA in Arabidopsis modulates enzyme activity in response to DNA damage. Genes Dev 26: 2512–2523. doi: 10.1101/gad.202960.112 PMID: 23109676
17. Romero DP, Blackburn EH (1991) A conserved secondary structure for telomerase RNA. Cell 67: 343–353. PMID: 1840508
18. Chen JL, Blasco MA, Greider CW (2000) Secondary structure of vertebrate telomerase RNA. Cell 100: 503–514. PMID: 10721988
19. Tzfati Y, Knight Z, Roy J, Blackburn EH (2003) A novel pseudoknot element is essential for the action of a yeast telomerase. Genes Dev 17: 1779–1788. PMID: 12832393
20. Chappell AS, Lundblad V (2004) Structural elements required for association of the *Saccharomyces cerevisiae* telomerase RNA with the Est2 reverse transcriptase. Mol Cell Biol 24: 7720–7736. PMID: 15314178
21. Qi X, Li Y, Honda S, Hoffmann S, Marz M, et al. (2013) The common ancestral core of vertebrate and fungal telomerase RNAs. Nucleic Acids Res 41: 450–462. doi: 10.1093/nar/gks980 PMID: 23093598
22. Cairney CJ, Keith WN (2008) Telomerase redefined: integrated regulation of hTR and hTERT for telomere maintenance and telomerase activity. Biochimie 90: 13–23. PMID: 17854971
23. Yi X, Tesmer VM, Savre-Train I, Shay JW, Wright WE (1999) Both transcriptional and posttranscriptional mechanisms regulate human telomerase template RNA levels. Mol Cell Biol 19: 3989–3997. PMID: 10330139
24. Box JA, Bunch JT, Tang W, Baumann P (2008) Spliceosomal cleavage generates the 3' end of telomerase RNA. Nature 456: 910–914. doi: 10.1038/nature07584 PMID: 19052544
25. Tang W, Kannan R, Blanchette M, Baumann P (2012) Telomerase RNA biogenesis involves sequential binding by Sm and Lsm complexes. Nature 484: 260–264. doi: 10.1038/nature10924 PMID: 22446625
26. Cifuentes-Rojas C, Kannan K, Tseng L, Shippen DE (2011) Two RNA subunits and POT1a are components of Arabidopsis telomerase. Proc Natl Acad Sci U S A 108: 73–78. doi: 10.1073/pnas.1013021107 PMID: 21164032
27. Beilstein MA, Brinegar AE, Shippen DE (2012) Evolution of the Arabidopsis telomerase RNA. Front Genet 3: 188. PMID: 23015808
28. Lysak MA, Koch MA, Beaulieu JM, Meister A, Leitch IJ (2009) The dynamic ups and downs of genome size evolution in Brassicaceae. Mol Biol Evol 26: 85–98. doi: 10.1093/molbev/msn223 PMID: 18842687
29. Beilstein MA, Nagalingum NS, Clements MD, Manchester SR, Mathews S (2010) Dated molecular phylogenies indicate a Miocene origin for *Arabidopsis thaliana*. Proc Natl Acad Sci U S A 107: 18724–18728. doi: 10.1073/pnas.0909766107 PMID: 20921408
30. Ziolkowski PA, Koczyk G, Galganski L, Sadowski J (2009) Genome sequence comparison of Col and Ler lines reveals the dynamic nature of Arabidopsis chromosomes. Nucleic Acids Res 37: 3189–3201. doi: 10.1093/nar/gkp183 PMID: 19305000
31. Samach A, Melamed-Bessudo C, Avivi-Ragolski N, Pietrokovski S, Levy AA (2011) Identification of plant RAD52 homologs and characterization of the *Arabidopsis thaliana* RAD52-like genes. Plant Cell 23: 4266–4279. doi: 10.1105/tpc.111.091744 PMID: 22202891
32. de Souza FS, Franchini LF, Rubinstein M (2013) Exaptation of transposable elements into novel cis-regulatory elements: is the evidence always strong? Mol Biol Evol 30: 1239–1251. doi: 10.1093/molbev/mst045 PMID: 23486611
33. Studer A, Zhao Q, Ross-Ibarra J, Doebley J (2011) Identification of a functional transposon insertion in the maize domestication gene tb1. Nat Genet 43: 1160–1163. doi: 10.1038/ng.942 PMID: 21946354
34. Sela N, Mersch B, Hotz-Wagenblatt A, Ast G (2010) Characteristics of transposable element exonization within human and mouse. PLoS One 5: e10907. doi: 10.1371/journal.pone.0010907 PMID: 20532223
35. Kelley D, Rinn J (2012) Transposable elements reveal a stem cell-specific class of long noncoding RNAs. Genome Biol 13: R107. doi: 10.1186/gb-2012-13-11-r107 PMID: 23181609
36. Johnson R, Guigo R (2014) The RIDL hypothesis: transposable elements as functional domains of long noncoding RNAs. RNA 20: 959–976. doi: 10.1261/rna.044560.114 PMID: 24850885
37. Kapusta A, Kronenberg Z, Lynch VJ, Zhuo X, Ramsay L, et al. (2013) Transposable elements are major contributors to the origin, diversification, and regulation of vertebrate long noncoding RNAs. PLoS Genet 9: e1003470. doi: 10.1371/journal.pgen.1003470 PMID: 23637635

38. Wutz A, Rasmussen TP, Jaenisch R (2002) Chromosomal silencing and localization are mediated by different domains of Xist RNA. Nat Genet 30: 167–174. PMID: 11780141

39. Cao J, Schneeberger K, Ossowski S, Gunther T, Bender S, et al. (2011) Whole-genome sequencing of multiple *Arabidopsis thaliana* populations. Nat Genet 43: 956–963. doi: 10.1038/ng.911 PMID: 21874002

40. Gazzani S, Gendall AR, Lister C, Dean C (2003) Analysis of the molecular basis of flowering time variation in Arabidopsis accessions. Plant Physiol 132: 1107–1114. PMID: 12805638

41. Clark MB, Johnston RL, Inostroza-Ponta M, Fox AH, Fortini E, et al. (2012) Genome-wide analysis of long noncoding RNA stability. Genome Res 22: 885–898. doi: 10.1101/gr.131037.111 PMID: 22406755

42. Schwanhausser B, Busse D, Li N, Dittmar G, Schuchhardt J, et al. (2011) Global quantification of mammalian gene expression control. Nature 473: 337–342. doi: 10.1038/nature10098 PMID: 21593866

43. Butelli E, Licciardello C, Zhang Y, Liu J, Mackay S, et al. (2012) Retrotransposons control fruit-specific, cold-dependent accumulation of anthocyanins in blood oranges. Plant Cell 24: 1242–1255. doi: 10.1105/tpc.111.095232 PMID: 22427337

44. Dutertre M, Lambert S, Carreira A, Amor-Gueret M, Vagner S (2014) DNA damage: RNA-binding proteins protect from near and far. Trends Biochem Sci 39: 141–149. doi: 10.1016/j.tibs.2014.01.003 PMID: 24534650

45. Rajagopalan R, Vaucheret H, Trejo J, Bartel DP (2006) A diverse and evolutionarily fluid set of microRNAs in *Arabidopsis thaliana*. Genes Dev 20: 3407–3425. PMID: 17182867

46. Wei W, Ba Z, Gao M, Wu Y, Ma Y, et al. (2012) A role for small RNAs in DNA double-strand break repair. Cell 149: 101–112. doi: 10.1016/j.cell.2012.03.002 PMID: 22445173

47. Lenzken SC, Loffreda A, Barabino SM (2013) RNA splicing: a new player in the DNA damage response. Int J Cell Biol 2013: 153634. doi: 10.1155/2013/153634 PMID: 24159334

48. Hu TT, Pattyn P, Bakker EG, Cao J, Cheng JF, et al. (2011) The *Arabidopsis lyrata* genome sequence and the basis of rapid genome size change. Nat Genet 43: 476–481. doi: 10.1038/ng.807 PMID: 21478890

49. Cheng F, Liu S, Wu J, Fang L, Sun S, et al. (2011) BRAD, the genetics and genomics database for Brassica plants. BMC Plant Biol 11: 136. doi: 10.1186/1471-2229-11-136 PMID: 21995777

50. Kannan K, Nelson AD, Shippen DE (2008) Dyskerin is a component of the Arabidopsis telomerase RNP required for telomere maintenance. Mol Cell Biol 28: 2332–2341. doi: 10.1128/MCB.01490-07 PMID: 18212040

51. Golisz A, Sikorski PJ, Kruszka K, Kufel J (2013) *Arabidopsis thaliana* LSM proteins function in mRNA splicing and degradation. Nucleic Acids Res 41: 6232–6249. doi: 10.1093/nar/gkt296 PMID: 23620288

52. Zhang X, Henriques R, Lin SS, Niu QW, Chua NH (2006) Agrobacterium-mediated transformation of *Arabidopsis thaliana* using the floral dip method. Nat Protoc 1: 641–646. PMID: 17406292

53. Pecinka A, Rosa M, Schikora A, Berlinger M, Hirt H, et al. (2009) Transgenerational stress memory is not a general response in Arabidopsis. PLoS One 4: e5202. doi: 10.1371/journal.pone.0005202 PMID: 19381297
